# Maternal immune-mediated conditions and ADHD risk in offspring

**DOI:** 10.1186/s12916-025-04227-3

**Published:** 2025-07-01

**Authors:** Kjersti Mæhlum Walle, Kristin Gustavson, Siri Mjaaland, Ragna Bugge Askeland, Per Magnus, Ezra Susser, W. Ian Lipkin, Camilla Stoltenberg, Michaeline Bresnahan, Ted Reichborn-Kjennerud, Mady Hornig, Helga Ask

**Affiliations:** 1 PsychGen Centre for Genetic Epidemiology and Mental Health, Norwegian Institute of Public Health, P. O. Box 222 Skøyen, Oslo, 0213 Norway; 2 Department of Psychology, University of Oslo, Oslo, Norway; 3 Department of Child Health and Development, Norwegian Institute of Public Health, Oslo, Norway; 4 Department of Immunology, Norwegian Institute of Public Health, Oslo, Norway; 5 Department of Medicine, University of Oslo, Oslo, Norway; 6 Centre for Fertility and Health, Norwegian Institute of Public Health, Oslo, Norway; 7 Department of Epidemiology, Columbia University Mailman School of Public Health, New York, NY USA; 8 New York State Psychiatric Institute, New York, NY USA; 9 Center for Infection and Immunity, Columbia University Mailman School of Public Health, New York, NY USA; 10 Departments of Neurology and Pathology, Columbia University Vagelos College of Physicians and Surgeons, New York, NY USA; 11 Department of Global Public Health and Primary Care, University of Bergen, Bergen, Norway; 12 NORCE Norwegian Research Centre, Bergen, Norway; 13 Feinstein Institutes for Medical Research, Northwell Health, New York, NY USA; 14 Promenta Research Center, University of Oslo, Oslo, Norway

**Keywords:** ADHD, Immune-mediated conditions, Pregnancy, MoBa, MBRN

## Abstract

**Background:**

Maternal immune-mediated conditions during pregnancy have been linked with increased risk of attention-deficit/hyperactivity disorder (ADHD) in offspring. The relative contributions of maternal inflammatory/immune responses versus shared genetic predispositions remain unclear. This study uses paternal immune-mediated conditions as a negative control to explore these factors, as we investigate associations between maternal immune-mediated conditions during pregnancy and offspring ADHD.

**Methods:**

Prospective data from the Norwegian Mother, Father, and Child Cohort Study (MoBa) was linked with the Medical Birth Registry of Norway (MBRN) and the Norwegian Patient Registry (NPR) to assess associations between prenatal exposure to maternal immune-mediated conditions and offspring ADHD risk up to age 18. Nationwide recruitment from 1999 to 2008 yielded 104,270 eligible mother–child pairs, with 21,340 children exposed to maternal allergic conditions (asthma, allergies, atopic conditions) and 7478 to other immune conditions (autoimmune, inflammatory). Paternal self-reported immune conditions served as negative controls. Children’s ADHD diagnoses were obtained from NPR, and Cox proportional hazard models estimated hazard ratios for ADHD.

**Results:**

Both overall categories were associated with increased offspring ADHD risk (allergic conditions HR 1.23, 95% CI, 1.14–1.34; other immune conditions HR 1.36, 95% CI, 1.21–1.53). Specific associations included maternal asthma (HR 1.47, 95% CI, 1.30–1.67), allergies (HR 1.20, 95% CI, 1.10–1.31), rheumatologic/musculoskeletal conditions (HR 1.64, 95% CI, 1.28–2.10), Crohn’s disease/ulcerative colitis (adjusted HR 1.95, 95% CI, 1.23–3.09), and endocrine conditions (HR 1.42, 95% CI, 1.15–1.77), specifically, type 1 diabetes (adjusted HR 2.50, 95% CI, 1.66–3.75). Although some paternal immune-mediated conditions (psoriasis, ulcerative colitis, Crohn’s disease) showed similar trends in ADHD risk, only paternal asthma was significantly associated (adjusted HR 1.26, 95% CI, 1.10–1.45).

**Conclusions:**

Several maternal immune-mediated conditions were associated with increased offspring ADHD risk. The higher, more consistent ADHD risk estimates with maternal conditions compared to paternal ones indicate that unmeasured genetic confounding does not fully explain these associations. These results suggest direct effects on fetal development through events at the maternal–fetal interface which may alter fetal immune responses and lead to greater ADHD risk in offspring. Asthma may be an exception to this mechanism, as paternal asthma was also linked with offspring ADHD risk.

**Supplementary Information:**

The online version contains supplementary material available at 10.1186/s12916-025-04227-3.

## Background

Attention-deficit hyperactivity disorder (ADHD) is a common childhood psychiatric disorder [1], with lifetime prevalence estimates by the age of 12 to be 5.4% among boys and 2.1% among girls in Norway [2]. Research into the origins of ADHD suggests a complex interplay of genetic and environmental factors [3], with most environmental factors still regarded as correlates [3]. Among these, prenatal environmental factors such as prematurity, low birthweight, and maternal stress and substance use during pregnancy have been identified [4–8]. Recent studies suggest that immune and inflammatory pathways, as well as infectious exposures, may play roles in the development of ADHD [9–11].

This study examines two broad categories of maternal immune-mediated conditions as risk factors for offspring ADHD: [1] asthma, allergy, and atopic conditions (hereafter: allergic conditions) and [2] autoimmune and inflammatory conditions, including urticaria, psoriasis, Crohn’s disease (CD), ulcerative colitis (UC), coeliac disease, rheumatoid arthritis (RA), ankylosing spondylitis (AS), systemic lupus erythematosus (SLE), fibromyalgia syndrome (FMS), type 1 diabetes (T1D), type 2 diabetes (T2D), gestational diabetes mellitus (GDM), and hyper/hypothyroidism (hereafter: other immune conditions). A central difference between these two broad categories is that allergic conditions involve exaggerated immune reactions to external triggers, while autoimmune and inflammatory conditions involve attack of the immune system on the body’s own tissues, leading to chronic, systemic immune dysregulation and inflammation [12].

Allergic and other immune conditions often co-occur in individuals and families [13–16], and mechanistic overlap exists, including activation of inflammatory cells and pathways [17]. However, variations in peripheral immune profiles, immune signaling pathways, cell types involved, and predominant immunoglobulin isotypes—such as immunoglobulin E (IgE) in allergic conditions versus immunoglobulin G (IgG) in inflammatory conditions [18–21]—suggest possible differing effects on fetal development. Therefore, different maternal immune-mediated conditions during pregnancy may also be differently associated with ADHD outcomes.

Only a few studies have investigated ADHD risk after prenatal exposure to maternal immune-mediated conditions, and the associations with allergic and other immune conditions have rarely been compared within the same population. Current findings suggest both categories of conditions to be associated with ADHD risk [22], possibly explained by immune processes happening during pregnancy [10, 23, 24]. The immune system’s cells and proteins are integral to neurodevelopment and functioning [25, 26], and there is evidence that maternal autoantibodies, such as IgG antibodies, can transfer across the placenta [27, 28], or transfer indirectly as shown with IgE antibodies [29]. These maternal immune alterations may impact fetal development through mechanisms like epigenetic modulation of neurodevelopmental gene expression, regulation of microglia activity, and alteration of synaptic functions [23, 24, 30].

Discrepancies in immune pathogenesis between allergic and other immune conditions may modify risk levels, phenotypic manifestations, or severity of ADHD outcomes. Even within the immune categories, the presence of different autoantibodies or differing targets of cellular autoimmune attack may contribute to diverse outcomes. By assessing a range of allergic and other immune conditions within the same study population, we aim to elucidate potential disparities in associations between different types or categories of immune-mediated disorders and ADHD risk.

The different types of diabetes are also distinct in their underlying mechanisms, suggesting potential differences in how maternal exposure may impact fetal development and influence risk for neurodevelopmental outcomes such as ADHD. Whereas type 1 diabetes (T1D) is characterized by an autoimmune response involving autoantibodies against insulin-producing beta cells [31], type 2 diabetes (T2D) and gestational diabetes mellitus (GDM) are linked to insulin resistance and low-grade inflammation, influencing the maternal metabolic state [32, 33]. This distinction warrants a separate analysis to more accurately assess impacts of autoimmune activation versus low-grade inflammation and metabolic influences on ADHD risk.

This study explores associations between maternal immune-mediated conditions and offspring ADHD in a sample of 104,270 pairs of mothers and children from the Norwegian Mother, Father, and Child Cohort (MoBa). By using paternal immune-mediated conditions as a negative control, we aim to discern whether associations arise primarily from maternal inflammatory or immune responses during pregnancy or shared genetic predispositions. The study aims to [1] estimate ADHD risk in offspring prenatally exposed to maternal immune-mediated conditions and [2] assess the impact of unmeasured confounding using paternal immune-mediated conditions as a negative control.

We hypothesize that maternal immune-mediated conditions during pregnancy increase the risk of ADHD in offspring, with differing impacts between different types of allergic and autoimmune/inflammatory conditions due to distinct immune and developmental pathways. Furthermore, we propose that the effect of maternal immune conditions on ADHD risk will be greater than paternal effects, highlighting the potential influence of environmental factors alongside genetic predispositions.

## Methods

### Study population and measures

MoBa is a population-based pregnancy cohort study including approximately 114,500 children, 95,200 mothers, and 75,200 fathers [34, 35]. Pregnant women from across Norway (1999–2008) were enrolled, with approximately 41% participation. The study uses quality-assured data, released for research in 2017 (v10), derived from maternal and paternal questionnaires completed at gestational weeks 17 and 30, as well as 6 months post-birth. MoBa data were linked to the Medical Birth Registry of Norway (MBRN), which holds comprehensive information on Norwegian births, including maternal diabetes, asthma, rheumatoid arthritis, age, parity, emigrations, and death records. The study was approved by The Regional Committee for Medical and Health Research Ethics (2014/2266).

**Fig. 1:**
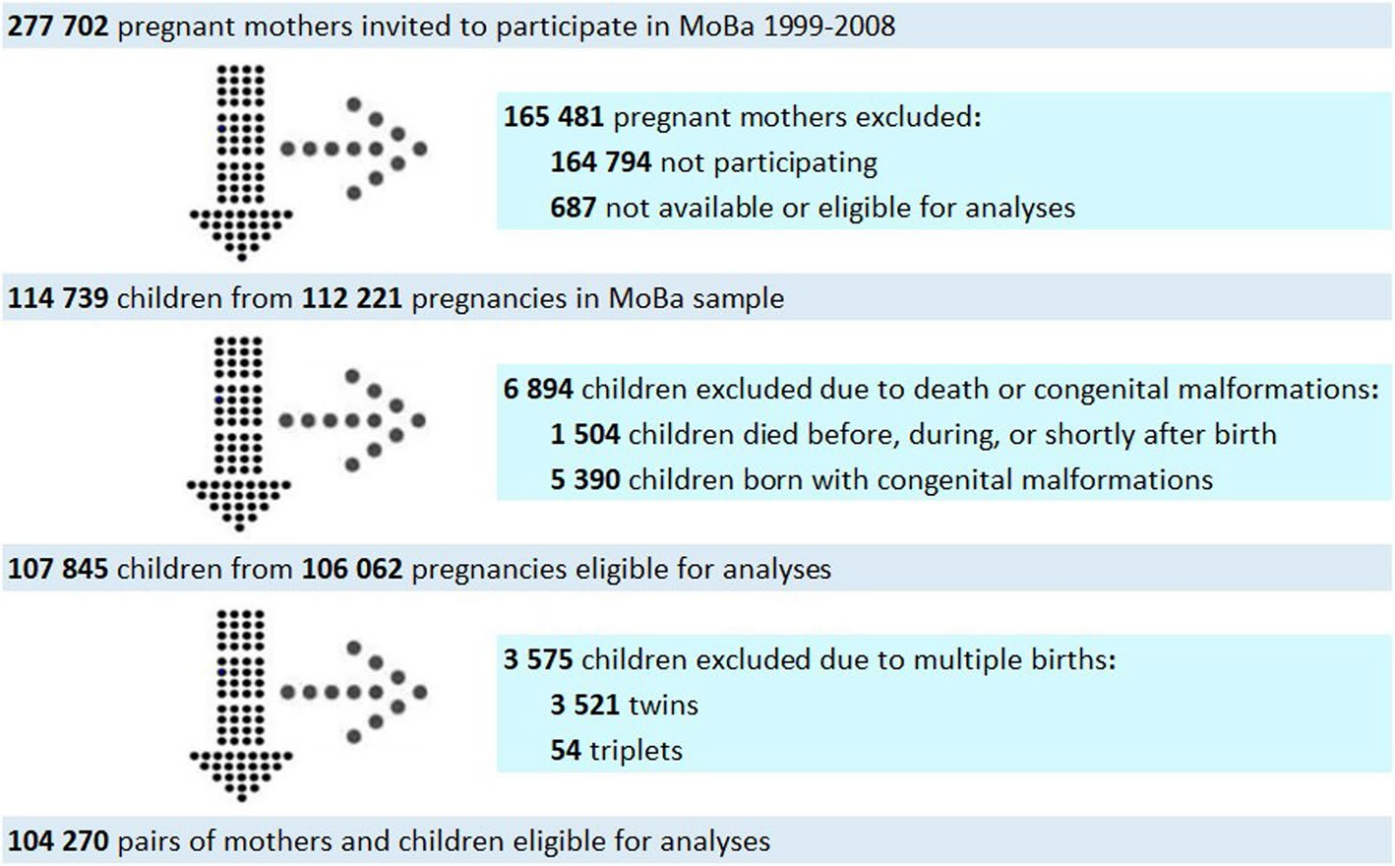
Flow chart of inclusion of participants. The flow chart shows the process of inclusion and exclusion of participants in the study. Reported are numbers of pregnant women invited to participate with their families, those who accepted and declined, those who were excluded for different reasons, and lastly how many were eligible for analysis

As low birth weight is associated with neonatal outcomes, and twins are more likely to be born with lower birth weight [36], children from multiple births were excluded from our sample. Other exclusion criteria included congenital malformations due to complexity of their etiologies, which may involve multiple genetic, environmental, and unknown factors [37]; death before the age of two; and unknown vital status (i.e., missing information on whether the child was alive or diseased at critical stages of the study timeline). Figure 1 displays numbers of participating and excluded mothers and children. The final study sample included 104,270 children with mothers and 71,344 fathers.

### Attention-deficit/hyperactivity disorder

Children’s ADHD diagnoses were gathered from the Norwegian Patient Registry (NPR), which includes information from government-funded clinics in Norway following the ICD-10 revision. Diagnoses were obtained for children with ADHD (F90 code) registered in the NPR between 2008 and 2017.

### Maternal and paternal immune-mediated conditions

Exposure variables were based primarily on parental self-report during pregnancy in MoBa questionnaires. Both parents reported their immune-mediated conditions by selecting from a list provided in a questionnaire (Table S1). Mothers also indicated if the condition occurred before and/or during pregnancy. To ensure clarity and avoid ambiguity in the variable categories, mothers reporting a specific immune-mediated condition before but not during pregnancy were excluded from the analyses related to that condition. However, exclusion from the analysis of a specific condition did not imply exclusion from the entire study. Additionally, there was information on three maternal immune-mediated conditions (diabetes, rheumatoid arthritis, and asthma) in MBRN, adding a few cases to our exposure variables for these conditions. As diagnoses were not person-identifiable in NPR prior to 2008, and most pregnancies occurred prior to this (1999–2008), we did not use NPR data to add cases in the exposure variables.

We categorized immune-mediated conditions into two groups: (1) asthma, allergy, and atopic conditions (allergic conditions) and (2) autoimmune and inflammatory conditions (other immune conditions). Further subcategories included (1a) asthma, (1b) allergies, (1c) atopic eczema, (1d) urticaria/hives, (2a) psoriasis, (2b) gastrointestinal conditions (Crohn’s disease (CD), ulcerative colitis (UC), coeliac disease), (2c) rheumatologic/musculoskeletal conditions (rheumatoid arthritis (RA), ankylosing spondylitis (AS), systemic lupus erythematosus (SLE), fibromyalgia syndrome (FMS)), and (2d) endocrine conditions (type 1 diabetes (T1D), hyper/hypothyroidism). We categorized exposure conditions based on affected organs or tissues to leverage the available data effectively, allowing us to group conditions with similar immunological pathways and physiological impacts. This categorization provides a structured framework to explore distinct immune responses and their potential differential effects on ADHD risk. By aligning our categories with the biological basis of the conditions, we aim to enhance the precision of our analyses, grounding our findings in relevant physiological mechanisms. In negative control designs, we focused on maternal exposure conditions that had corresponding data collected from fathers, ensuring comparability between maternal and paternal information despite slight differences in the questionnaires. Due to the absence of queries regarding paternal thyroid conditions and restriction of paternal report to unspecified diabetes types, the negative control analysis for the endocrine category assessed overall diabetes for comparability between maternal and paternal exposure groups (Table S1). Using maternal data, we further investigated how exposure to different types of diabetes in pregnant mothers affected the risk of ADHD in offspring.

### Covariates

To ensure that covariate selection was rooted in existing knowledge of relevant causal pathways, we first selected potential covariates based on previous research investigating maternal immune-mediated conditions as ADHD risk factors [22, 38–42] and available data. For each analysis we planned to conduct, covariates were evaluated for associations with both exposure and ADHD outcome to identify potential confounding. Directed acyclic graphs (DAGs) are effective tools for exploring complex causal relationships because they help clarify and visually represent pathways between variables [43]. This can prevent over-adjustment or unnecessary inclusion of covariates that do not contribute additional control, thereby reducing the risk of introducing collider bias or overfitting models [43]. We used Dagitty models [44] to create DAGs and define minimal sufficient adjustment sets of covariates for each specific analysis. Information on covariates selected is available in Table 1 (selection process details in Additional file 1: Tables S2–S4 and Figs. S1–S10; handling of missing data described in Additional file 1).

**Table 1:** Descriptive statistics for covariates

	Total sample, *n* = 104,270		
Birth year, *n*, mean, [SD]	104,270	2005	[2.217]
Mothers’ age, *n,* mean, [SD]	104,270	30.1	[4.666]
Mothers’ ADHD symptoms score, *n*, mean, [SD]	52,947	2.1	[0.576]
Missing data on mothers’ ADHD symptoms scores, *n,* (%)	51,322		(49.22)
Highest level of education among parents, *n,* (%)			
Less than high school graduate	3495		(3.35)
High school graduate	23,250		(22.30)
Undergraduate education completed	34,055		(32.66)
Postgraduate education (master or doctorate) completed	28,754		(27.58)
Missing data on level of education	14,715		(14.11)
Parents’ relationship status, *n,* (%)			
Married or in a relationship	90,754		(87.04)
Single	3181		(3.05)
Missing data on parents’ relationship status	10,334		(9.91)
Parity, *n,* (%)			
First born	45,545		(43.68)
Second born	37,678		(36.14)
Third (or more) born	21,046		(20.18)
Mother’s smoking habits, *n,* (%)			
Mothers smoking before pregnancy	26,326		(25.25)
Mothers not smoking before pregnancy	66,269		(63.56)
Missing data on smoking habits	11,674		(11.20)
Alcohol use before pregnancy, *n,* (%)			
Never	6555		(6.29)
Less than 3 units per month	57,620		(55.26)
1–3 units per week	22,489		(21.57)
4–7 units per week	948		(0.91)
Missing data on alcohol use	16,657		(15.98)
The mother’s previous mental disorders, *n,* (%)			
Previously diagnosed with anorexia, bulimia, or other eating disorders, depression, or anxiety	19,264		(18.48)
Never diagnosed with anorexia, bulimia, or other eating disorders, depression, or anxiety	85,005		(81.52)

### Statistical analysis

Analyses were performed using SPSS version 27 and Stata version 17.

Crude and adjusted hazard ratios (HRs and aHRs) for ADHD with 95% confidence intervals (CIs) were estimated using Cox proportional hazard models. Separate analyses were conducted for each overall group and subgroups. The child’s age served as time variable, and follow-up started on the child’s third birthday, concluding with either an ADHD diagnosis, emigration, death, or by December 31st, 2017, whichever occurred first. Children were followed up until age 8–18 years. In our analysis, comparisons for one immune condition exposure (present/absent) included children who may have also been exposed to other immune conditions.

To separate the effects of maternal immune-mediated responses during pregnancy from shared genetic factors, a negative control strategy was utilized. Previous studies have conducted negative control analyses by comparing outcomes of maternal exposures during pregnancy with paternal exposures or those of other relatives [7, 45]. This approach tested associations between paternal immune-mediated conditions—which are not expected to directly impact the fetal environment beyond genetic/epigenetic effects—and offspring ADHD risk. Both maternal and paternal analyses are subject to similar confounding factors; however, except for paternal epigenetic influences and potential genetic effects on the placental environment [46], maternal conditions predominantly affect the gestational milieu. Stronger associations with maternal immune-mediated conditions compared to paternal ones suggest an influence of maternal immune-mediated responses during pregnancy. Conversely, equal maternal and paternal associations imply that shared genetic and confounding factors are likely explanations. To prevent any bias that might occur if an observed association for one parent was driven by correlated conditions in the other parent, maternal and paternal associations were mutually adjusted for each other, as well as for the minimal sufficient adjustment sets of covariates.

To address multiple testing—five tests within each family of tests (allergic conditions and other immune conditions)—we adjusted the alpha level to 0.01.

Finally, sensitivity analyses examined the potential impact of folate use during pregnancy, recognizing its role in immune system balance [47]. Sensitivity analyses assessing effects of medical treatments were also performed.

## Results

### Descriptives

Table 1 shows descriptive statistics for covariates. Among the children, 3600 were diagnosed with ADHD (3.5%).

### Overall categories: allergic and other immune conditions

Table 2 presents the risk estimates for the overall categories of maternal immune-mediated conditions. Both categories showed increased ADHD risk: allergic conditions (aHR = 1.23, CI: 1.14, 1.34) and other immune conditions (aHR = 1.36, CI: 1.21, 1.53). These findings suggest a broad impact of maternal immune health on offspring ADHD risk.

**Table 2:** Associations between maternal immune-mediated conditions during pregnancy and ADHD in offspring examined with Cox proportional hazard analyses

		No. of person-months at observed risk^b^	Incidence rate^c^	Crude				Adjusted^a^			
				Hazard ratio	SE	95% CI	*P*	Hazard ratio	SE	95% CI	*P*
Asthma/allergic/atopic conditions	No	10,016,598	22.6	Ref				Ref			
	Yes	3,106,811	27.0	1.20	0.05	1.11–1.30	< 0.001	1.23	0.05	1.14–1.34	< 0.001
Autoimmune/inflammatory conditions	No	13,948,303	23.0	Ref				Ref			
	Yes	1,078,983	29.9	1.31	0.08	1.17–1.47	< 0.001	1.36	0.08	1.21–1.53	< 0.001
Asthma	No	13,939,521	22.6	Ref				Ref			
	Yes	743,603	36.3	1.61	0.1	1.42–1.83	< 0.001	1.47	0.09	1.30–1.67	< 0.001
Any allergy (pollen, animal, other)	No	11,181,584	23.3	Ref				Ref			
	Yes	2,385,555	26.3	1.14	0.05	1.04–1.24	0.004	1.20	0.05	1.10–1.31	< 0.001
Atopic eczema	No	14,248,317	23.6	Ref				Ref			
	Yes	548,285	24.8	1.06	0.09	0.89–1.26	0.528	1.13	0.10	0.95–1.34	0.176
Urticaria/hives	No	14,625,830	23.6	Ref				Ref			
	Yes	110,415	26.3	1.11	0.21	0.77–1.60	0.584	1.11	0.21	0.77–1.60	0.574
Psoriasis	No	14,815,126	23.6	Ref				Ref			
	Yes	239,105	28.0	1.19	0.15	0.93–1.51	0.162	1.14	0.14	0.89–1.45	0.296
Gastrointestinal conditions	No	15,052,760	23.7	Ref				Ref			
	Yes	108,500	27.6	1.20	0.23	0.83–1.73	0.340	1.28	0.24	0.89–1.85	0.189
Rheumatologic/musculoskeletal conditions	No	14,966,233	23.4	Ref				Ref			
	Yes	154,198	42.2	1.80	0.23	1.41–2.31	< 0.001	1.64	0.21	1.28–2.10	< 0.001
Endocrine conditions	No	14,609,045	23.4	Ref				Ref			
	Yes	298,589	30.5	1.32	0.14	1.07–1.63	0.011	1.42	0.15	1.15–1.77	0.001

Separate analyses were performed for each of the exposure variables. *CI*, confidence interval; *Ref*, reference group to which mothers with immune-mediated disorders are compared. The *α* level was set to 0.01 to indicate significant associations

^a^Each analysis used specific adjustment sets of covariates: asthma/allergic/atopic conditions: child’s birth year, mother’s parity, alcohol use before pregnancy, previous mental disorders, and self‐reported ADHD symptoms; autoimmune/inflammatory conditions: child’s birth year, parental relationship status, mother’s age, parity, smoking and alcohol use before pregnancy, previous mental disorders, and self‐reported ADHD symptoms; asthma: parental relationship status, mother’s age, parity, smoking and alcohol use before pregnancy, previous mental disorders, and self‐reported ADHD symptoms; any allergy: child’s birth year, parental educational attainment, mother’s age, parity, smoking and alcohol use before pregnancy, previous mental disorders, and self‐reported ADHD symptoms; atopic eczema: child’s birth year, parental educational attainment, mother’s parity, alcohol use before pregnancy, previous mental disorders, and self‐reported ADHD symptoms; urticaria/hives: parental educational attainment, mother’s parity, and alcohol use before pregnancy; psoriasis: parental educational attainment and relationship status, mother’s smoking, and previous mental disorders; gastrointestinal conditions: child’s birth year, mother’s age, and previous mental disorders; rheumatologic/musculoskeletal conditions: parental educational attainment and relationship status, mother’s parity, smoking and alcohol use before pregnancy, and self‐reported ADHD symptoms; endocrine conditions (T1D and hyper/hypothyroidism): child’s birth year, parental educational attainment, mother’s age, parity, smoking and alcohol use before pregnancy, and self‐reported ADHD symptoms

^b^Observation period between January 2008 and December 2017 for participants born in or before January 2008

^c^Per 100,000 person-months under observed risk

### Asthma

**Fig. 2:**
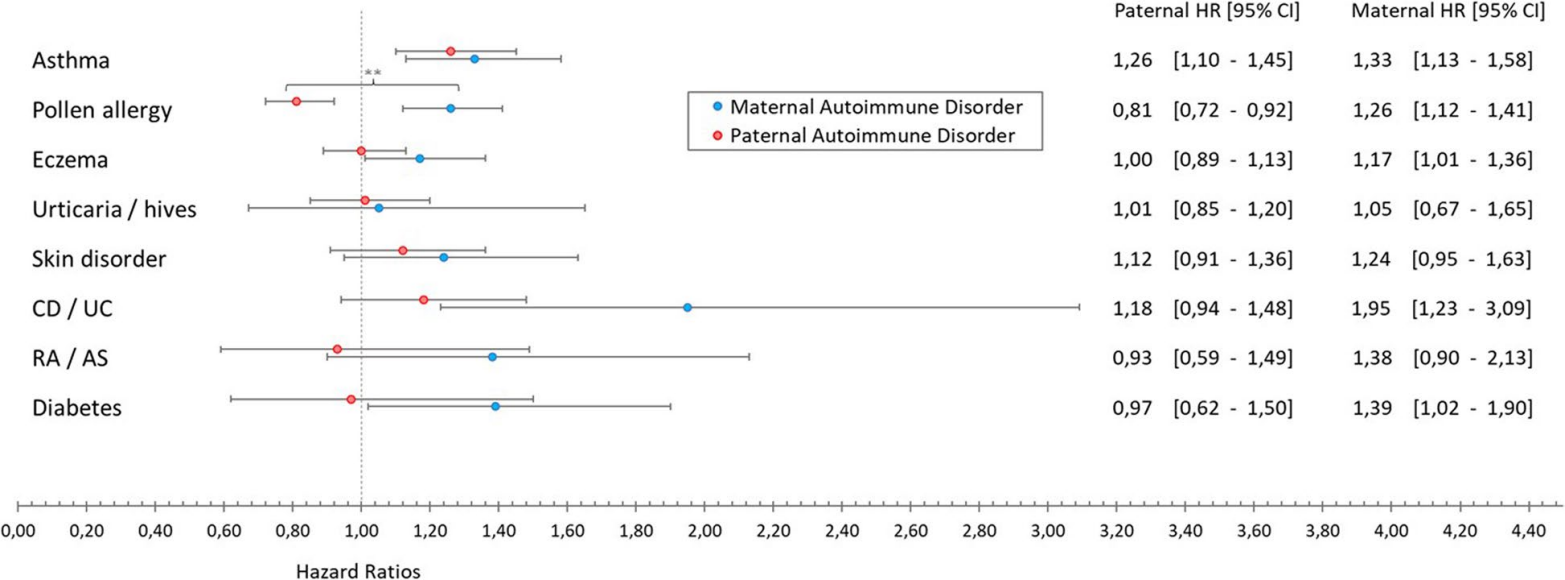
Forest plot comparing hazard ratios for ADHD after maternal and paternal immune-mediated conditions. The forest plot shows the hazard ratios for offspring ADHD when children were prenatally exposed to maternal immune-mediated conditions as compared to when their fathers had the same conditions. Risk estimates for ADHD were higher and more consistent after prenatal exposure to maternal conditions compared to those of paternal conditions. Maternal and paternal immune-mediated conditions were mutually adjusted for each other as well as for the minimal sufficient adjustment sets of covariates selected for each specific condition

Asthma emerged as a significant factor in increasing ADHD risk. As indicated in Table 2, maternal asthma was associated with a substantial risk increase (aHR = 1.47, CI: 1.30, 1.67). The negative control analysis in Fig. 2 pointed to a similar pattern with paternal asthma (aHR = 1.26, CI: 1.10, 1.45), underscoring the importance of asthma in both maternal and paternal histories.

### Allergies

In examining allergies, and as can be seen in Table 2, we found that any maternal allergy increased ADHD risk (aHR = 1.20, CI: 1.10, 1.31). Interestingly, Fig. 2 reveals contrasting effects of maternal and paternal pollen allergies, with maternal pollen allergies linked to elevated risk (aHR = 1.26, CI: 1.12, 1.41), whereas paternal pollen allergies suggested a preventive effect (aHR = 0.81, CI: 0.72, 0.92). This difference between maternal and paternal exposure was statistically significant (X2 (df = 1, *N* = 64,167) = 26.49, *P* < 0.001).

### Gastrointestinal conditions

Maternal gastrointestinal conditions overall (including the conditions Crohn’s disease (CD), ulcerative colitis (UC), and coeliac disease) did not reveal a significant association; however, the negative control analysis that specifically assessed Crohn’s disease and ulcerative colitis (CD/UC) revealed significant effects of maternal CD/UC (aHR = 1.95, CI: 1.23, 3.09), but not of paternal CD/UC. Figure 2 shows that the hazard ratios for maternal versus paternal CD/UC were quite high; however, confidence intervals were wide, and the statistical difference only approached significance (X2 (df = 1, *N* = 70,820) = 3.75, *P* = 0.053).

### Rheumatologic/musculoskeletal conditions

The overall category of maternal rheumatologic/musculoskeletal conditions (including the conditions rheumatoid arthritis (RA), ankylosing spondylitis (AS), systemic lupus erythematosus, and fibromyalgia syndrome) was in the initial analysis associated with increased ADHD risk (aHR = 1.64, CI: 1.28, 2.10). However, the exposure in the negative control analysis was limited to assess the conditions RA and AS and showed a similar trend for maternal exposure but not for paternal exposure.

### Endocrine conditions and diabetes

Maternal endocrine conditions (including type 1 diabetes (T1D) and thyroid conditions) showed an increased risk of offspring ADHD (aHR = 1.42, CI:1.15, 1.77). The negative control analysis, investigating any type of diabetes, displayed an effect of maternal diabetes (aHR = 1.39, 95% CI: 1.02, 1.90) but not one of paternal diabetes. Analyzing type 1 diabetes (T1D), type 2 diabetes (T2D), and gestational diabetes mellitus (GDM) separately (mothers only), offspring ADHD risk increased only with maternal T1D (aHR 2.50, 95% CI:1.66–3.75) (Table 3). No significant associations were noted for maternal type 2 diabetes (T2D) and gestational diabetes mellitus (GDM).

**Table 3:** Associations between different types of maternal diabetes in pregnancy and ADHD in offspring examined with Cox proportional hazard analyses

		No. of person-months at observed risk^b^	Incidence rate^c^	Crude				Adjusted^a^			
				Hazard ratio	SE	95% CI	*P*	Hazard ratio	SE	95% CI	*P*
Diabetes 1	No	14,972,880	23.5	Ref				Ref			
	Yes	43,263	57.8	2.48	0.51	1.66–3.73	< 0.001	2.5	0.52	1.66–3.75	< 0.001
Diabetes 2	No	14,972,880	23.5	Ref				Ref			
	Yes	21,993	22.7	0.98	0.22	0.64–1.52	0.944	0.98	0.22	0.64–1.52	0.940
Gestational diabetes mellitus	No	14,972,880	23.5	Ref				Ref			
	Yes	140,489	27.8	1.05	0.04	0.97–1.13	0.259	1.04	0.04	0.96–1.12	0.341

Separate analyses were performed for each type of diabetes. *CI*, confidence interval; *Ref*, reference group to which mothers with diabetes are compared. The *α* level was set to 0.01 to indicate significant associations

^a^Analyses are adjusted for the following covariates: child’s birth year, parental educational attainment, mother’s age, parity, smoking and alcohol use before pregnancy, and self‐reported ADHD symptoms

^b^Observation period between January 2008 and December 2017 for participants born in or before January 2008

^c^Per 100,000 person-months under observed risk

### Sensitivity analyses

Sensitivity analyses (Additional file 1: Tables S9 and S10) found no interactions between folic acid and specific conditions, or any effects of medical treatments on offspring ADHD risk.

## Discussion

Our findings suggest that maternal immune-mediated conditions, both allergic and other immune conditions, are associated with a higher risk of ADHD in offspring. Specifically, maternal asthma is associated with a 47% higher risk, allergies with a 20% higher risk, rheumatologic/musculoskeletal conditions with a 64% higher risk, and endocrine conditions with a 42% higher risk. When examining a smaller sample with information on both paternal and maternal conditions available, asthma was the only paternal condition linked to an increased ADHD risk in offspring, showing a 26% higher risk. In comparison, for maternal conditions increased risk of ADHD in offspring was found with asthma (33% higher risk), pollen allergies (26% higher risk), Crohn’s disease/ulcerative colitis (CD/UC) (95% higher risk), and any type of diabetes (39% higher risk). Notably, the difference in risk between maternal and paternal conditions was only significant for pollen allergies, where maternal and paternal associations showed opposing directions. Comparison of maternal diabetes subtypes revealed that type 1 diabetes was associated with a 150% higher risk of ADHD in offspring, while type 2 diabetes and gestational diabetes mellitus were not significantly associated with ADHD risk. This underscores the role of type 1 diabetes in the observed association between any maternal diabetes and ADHD risk. Information on the diabetes type was not available for fathers, limiting our analysis of paternal diabetes.

This study consistently found higher ADHD risk associated with maternal immune-mediated conditions compared to paternal ones, particularly allergies, which showed significant directional differences. While maternal conditions uniformly showed trends toward increased ADHD risk, paternal conditions exhibited more variability, with only asthma showing a significant association. Previous research has indicated higher ADHD risk after exposure to maternal, compared to paternal, autoimmune and atopic disorders, with similar findings regarding risk of autism spectrum disorder (autism) [48], suggesting potential maternal-specific immune mechanisms during pregnancy.

### Maternal immune activation and fetal development

Maternal exposure to immune-mediated conditions during pregnancy could heighten offspring ADHD risk through mechanisms involving maternal immune activation, likely impacting fetal development via the placenta [49]. Research on “fetal programming” underscores the placenta’s significance as the first functional organ of the fetus, facilitating maternal–fetal cellular interactions [24, 50–53], which may influence fetal immune system development [50]. Disruptions in these interactions may potentially contribute to neuropsychiatric conditions like ADHD and autism [54, 55]. Studies associating neurodevelopmental conditions and traits with prenatal exposure to maternal antibodies [56] suggest that maternal immune-mediated conditions may impact neurodevelopment through antibody-mediated pathways, including potential transference across the placenta and placental cytokine expression. Different maternal immune conditions can uniquely impact fetal development through a range of mechanisms, some of which overlap while others are distinct, as detailed in the following sections. These mechanisms encompass aspects, such as immune activation, response shifts, antibody transfer, metabolic influences, dopaminergic system interactions, and genetic and epigenetic factors.

### Maternal immune response shifts during pregnancy

During normal pregnancies, the maternal immune response shifts from Th1 (cell-mediated) to Th2 (humoral) dominance [49], reducing inflammatory cytokine production, while increasing regulatory T-cell (Treg) production [49, 57]. This shift can have varying implications for maternal immune-mediated conditions. Atopic conditions, such as asthma and allergies, are typically Th2-dominant, and the enhanced Th2 response during pregnancy could exacerbate these conditions due to increased humoral activity [58]. Conversely, autoimmune conditions (such as RA, CD, UC) that are predominantly Th1-mediated may experience symptom improvement during pregnancy, as the Th2 shift downregulates typical inflammatory pathways [59]. However, this shift in maternal immune response also results in elevated anti-inflammatory cytokine levels in maternal blood [49], which again may influence brain development pathways. Evidence from animal models suggests that maternal immune activation may reduce the accumulation of Tregs at the maternal–fetal interface, and that reversing this may reduce adverse neurodevelopmental outcomes [60]. The mechanism by which maternal immune molecules influence fetal immune system and neurodevelopment, therefore, remains uncertain [24].

The placenta may play a pivotal role in modulating these effects. It contains its own macrophages, Hofbauer cells, producing various cytokines and chemokines [61]. In response to maternal inflammation, the placenta may release cytokines and chemokines into fetal circulation, potentially affecting ongoing fetal growth and neurodevelopment [49, 62, 63]. Cytokine release and subsequent inflammation are also key factors in altering dopaminergic systems, a feature strongly associated with ADHD.

Moreover, placental inflammation can activate microglia [49], immune cells essential for neurodevelopmental processes, including axon guidance and synaptic pruning [64, 65]. Activated excessively, microglia can release pro-inflammatory cytokines and proteins, potentially harming neurons and disrupting neurodevelopment [66, 67].

Neurodevelopmental effects may also be mediated by activation of placental Toll-like receptors, which respond to various environmental threats [68].

Since Th2 dominance during pregnancy may result in exaggerated anti-inflammatory responses that intensify atopic conditions such as asthma and allergies [58], this heightened immune activity could amplify its influence on fetal development, potentially altering neurodevelopmental pathways and increasing ADHD risk. Autoimmune conditions characterized by Th1 dominance may experience symptom improvement during pregnancy due to reduced inflammation [59], potentially diminishing the fetal risk associated with maternal exposure to these conditions [58].

### Placental antibody transfer

Initially, only IgG antibodies were believed to cross the placenta, typically from the 13th week of gestation [69]. IgG autoantibodies play a significant role in autoimmune and inflammatory conditions, such as rheumatoid arthritis (RA), ankylosing spondylitis (AS), systemic lupus erythematosus (SLE), and fibromyalgia syndrome (FMS) [21, 70, 71], and this study found associations between maternal rheumatologic musculoskeletal conditions, especially SLE and FMS, and ADHD risk. Maternal SLE has previously been linked to increased risk of neurodevelopmental disorders or other developmental challenges, particularly in boys [72], possibly due to placental transfer of maternal IgG autoantibodies [73, 74]. Similarly, transferring IgG autoantibodies from FMS patients to mice has been shown to induce sensory hypersensitivity, suggesting a potential mechanism for maternal FMS impacting offspring neurodevelopment [71]. While placental transfer of IgG antibodies is long recognized, recent evidence suggests that IgE autoantibodies can also be transferred across the placenta, though indirectly through immune complex linkage [27–29]. Atopic conditions like asthma and allergies often involve elevated IgE levels [21]. Maternal asthma and allergies were associated with offspring ADHD risk, and effect estimates were higher than for paternal exposure. Elevated IgE and resulting inflammation may contribute to irregularities in fetal brain development. The trend of higher ADHD risk after prenatal exposure to maternal eczema only approached significance, which could be due to the lack of differentiation between intrinsic and extrinsic types of eczema, which are associated with different levels of IgE [75]. On the other hand, another study that measured prenatal IgE did not find an association with offspring ADHD outcomes [76], which underscores the need for further research.

### Dopaminergic system and immune interaction

Impairments in the dopaminergic system are recognized as a significant mechanism in ADHD [77, 78], with studies linking genes like dopamine receptor D4 and the dopamine transporter to the disorder [79]. Dopamine receptors on immune cells suggest dopamine involvement in immune-inflammatory responses [80]. Dysregulation of peripheral dopamine levels is linked to rheumatoid arthritis and inflammatory bowel disease [80, 81]. Alterations in dopamine pathways due to maternal immune conditions could contribute to the increased ADHD risk observed in offspring. Maternal immune activation in rats affects offspring dopaminergic signaling [62], and, given dopamine’s role in ADHD [77] and immune disorders [62, 80], dopamine dysregulation presents a common mechanism and plausible link. Our study supports this, showing increased ADHD risk with maternal rheumatologic/musculoskeletal conditions and maternal CD/UC.

### Diabetes and ADHD risk—autoimmune inflammation vs metabolic influences

We observed a strong association between maternal type 1 diabetes (T1D) and the risk of ADHD in offspring, consistent with previous research [22, 38, 40–42]. Despite few maternal T1D cases in the diabetes group (*N* = 301 of 1425), analyzed separately, maternal T1D HR was notably high (HR = 2.5). In contrast, other diabetes types such as maternal type 2 diabetes (T2D) and gestational diabetes mellitus (GDM) showed no individual associations with offspring ADHD risk.

Unfortunately, fathers did not specify diabetes type, limiting direct comparison of maternal and paternal T1D exposure. Instead, the broader exposure category “any diabetes” was compared between mothers and fathers. Any maternal diabetes exposure had higher HRs than any paternal diabetes, though not significantly different. This could be due to the inclusion of all diabetes types causing group heterogeneity.

GDM, emerging during pregnancy [82], shares features like insulin resistance with T2D and lacks T1D’s autoimmune aspect [33]. While T1D involves autoantibodies against insulin-producing cells [31], T2D’s immune role centers on low-grade inflammation and insulin resistance [32, 33]. The significant increase in offspring ADHD risk with maternal T1D, and not with other diabetes types, may be explained by the autoimmune inflammation impacting fetal neural development, in contrast to the metabolic influences of other diabetes types.

### Genetic and epigenetic influences

Maternal and paternal asthma were both linked to offspring ADHD, aligning with recent cohort studies in Denmark and Taiwan [48, 83]. A recent meta-analysis also reported a phenotypic association between ADHD and asthma, suggesting shared familial factors (genetic liability and/or shared environmental factors) contributing to their risk [84].

ADHD and many immune-mediated conditions have heritable components [85–87] and their high comorbidity [38, 88] suggest shared genetic variations. Genetic and epigenetic factors, influencing gene expression timing and location, and possibly influenced by prenatal environment [89], are implicated in autoimmune and atopic disorder development [90]. These genetic and epigenetic changes could affect brain development processes, contributing to ADHD expression. Research suggests that epigenetic modifiers affecting DNA methylation (DNAm) and histone remodeling are crucial for normal neurodevelopment [91]. DNAm is extensively studied as an epigenetic marker of ADHD [89], due to its role in brain maturation and function [92], susceptibility to genetic and environmental influences [93, 94], and links to various health issues, including immune-mediated conditions [95] and psychiatric disorders [91].

### Strengths and limitations

This study has several strengths. Data were drawn from an extensive birth cohort, facilitating detection of nuanced differences in ADHD risk across various groups and allowing for comparison between maternal gestational exposure and paternal exposure, offering insights into the complex interplay of environmental and genetic factors. Prospective data collection spanned multiple pregnancy exposures, with 8–18 years of follow-up. Additionally, the alpha level was adjusted to mitigate the risk of false positives.

The study has limitations that could impact generalizability. Firstly, the 41% participation rate in MoBa could introduce selection bias, as participants are typically healthier and better educated than the overall population [96, 97]. However, previous research found associations between various exposures and outcomes in MoBa participants and the total population to be comparable [96, 97]. Children with ADHD in MoBa show similar functioning and psychosocial challenges as those in the broader Norwegian population [98]. Simulations suggest that associations between risk factors and health outcomes remain robust even with underrepresented groups in the sample [99]. Secondly, the oldest children in the sample may have received ADHD diagnoses before individual diagnoses were recorded in NPR, potentially resulting in false negatives if they only sought specialist healthcare in their early years (< 2008). Thirdly, self-reported medical conditions may be biased. Men with symptoms similar to women may receive more thorough treatment [100], potentially affecting diagnosis rates. Moreover, unclear distinctions between immune disease types and diagnosis delays [101] can impact report accuracy. Reliable research depends on accurate data collection; misclassification of cases can dilute the effects. Fourthly, there is a significant amount of missing data for maternal ADHD symptoms, with approximately 49% missing. Although we used multiple imputation to address this issue, it is possible that the data missingness is not random, as ADHD symptoms could affect the likelihood of completing questionnaires. The results should therefore be interpreted with this consideration in mind. Fifthly, we acknowledge that some covariates might not have shown significant associations due to measurement imperfections or sample-specific characteristics, potentially leading to their exclusion despite theoretical relevance. Also, ~ 70% of fathers lacked information regarding ADHD symptoms. Consequently, we did not include paternal ADHD symptoms as a covariate, though this could be a potential confounder, particularly between paternal immune-mediated conditions and offspring ADHD. However, significant associations between maternal immune-mediated conditions and offspring ADHD persisted after adjusting for maternal ADHD symptoms. Conversely, associations with paternal conditions were weaker, even without adjusting for paternal ADHD symptoms. Sixthly, comparisons made for one immune condition exposure could include children exposed to additional immune conditions, which complicates the isolation of the specific impact of each condition. Seventhly, our data on asthma is not specific with regard to atopy. Eighthly, in analyses comparing maternal gestational immune-mediated conditions with paternal immune-mediated conditions, paternal influence during gestation cannot be discounted, given animal studies indicating the paternal genome may impact placental development through genomic imprinting [102]. Lastly, maternal medical treatment during gestation could affect associations. However, sensitivity analyses revealed no interactions between medical treatments and conditions.

## Conclusions

This large population-based cohort revealed increased offspring ADHD risk when mothers had certain immune-mediated conditions during pregnancy. Paternal asthma was also associated with offspring ADHD, implying some shared mechanisms involving genetic/epigenetic influences established at the time of conception. Maternal conditions may additionally have direct effects on fetal development through the maternal–fetal interface, potentially altering immune responses and increasing ADHD risk. The complex interplay of genetic/epigenetic, immune, environmental, and placental factors likely contributes to these associations. Further research is needed to explore the specific genetic and epigenetic pathways, as well as to identify precise immune and environmental factors that may mediate these risks. Longitudinal studies incorporating advanced biomarker analyses and detailed environmental exposure assessments could provide more comprehensive insights into the causal pathways and potential intervention points. Additionally, investigating whether these findings are consistent across diverse populations and settings would enhance the generalizability of our conclusions.

## Supplementary Information

Additional file 1: This file includes details on exposure data (Table S1), covariate selection and associations between covariates and between covariates and exposure variables (Tables S2 and S3), covariates included in different Dagitty models (DAGs) (Table S4), DAGs that forms the basis of the minimal sufficient adjustment sets of covariates for each of the exposure categories (Figs. S1–S10), and information regarding the handling of missing data. In addition, this contains descriptive information on exposed and unexposed cases as well as missing data for the main analyses (Tables S5 and S6). Additional analyses conducted on unimputed data are also included in the file (Table S7), as well as more detailed statistics from the negative control analyses (Table S8) and results from the sensitivity analyses (Tables S9 and S10). Table S1 Health conditions reported in questionnaires at specific times during pregnancy. Table S2 Associations between considered covariates and exposure variables. Table S3 Associations between covariates. Table S4 Covariates included in Dagitty models and minimum sufficient adjustment sets for each specific analysis. Table S5 Descriptive statistics for dataset analyzing maternal immune-mediated conditions in pregnancy and ADHD in offspring with Cox proportional hazards. Table S6 Descriptive statistics for dataset analyzing maternal diabetes in pregnancy and ADHD in offspring with Cox proportional hazards. Table S7 Associations between maternal immune-mediated conditions during pregnancy and ADHD in offspring examined with Cox proportional hazard analyses. Table S8 Associations between maternal gestational immune-mediated conditions and offspring ADHD diagnoses compared with the associations with paternal immune-mediated conditions, mutually adjusted for each other. Table S9 Sensitivity analyses for use of folic acid between 4 weeks before pregnancy and 8 weeks of gestation. Table S10 Analyses stratified by the specific immune-mediated condition assessing whether medications alters the risk of developing ADHD among, e.g., asthmatics. Fig. S1 Directed acyclic graph (DAG) for covariate selection in analyses on prenatal exposure to asthma, allergy, and atopic conditions and ADHD risk. Fig. S2 DAG for covariate selection in analyses on prenatal exposure to autoimmune and inflammatory conditions and ADHD risk. Fig. S3 DAG for covariate selection in analyses on prenatal exposure to asthma and ADHD risk. Fig. S4 DAG for covariate selection in analyses on prenatal exposure to allergies and ADHD risk. Fig. S5 DAG for covariate selection in analyses on prenatal exposure to eczema and ADHD risk. Fig. S6 DAG for covariate selection in analyses on prenatal exposure to urticaria/hives and ADHD risk. Fig. S7 DAG for covariate selection in analyses on prenatal exposure to psoriasis and ADHD risk. Fig. S8 DAG for covariate selection in analyses on prenatal exposure to gastrointestinal conditions and ADHD risk. Fig. S9 DAG for covariate selection in analyses on prenatal exposure to rheumatologic musculoskeletal conditions and ADHD risk. Fig. S10 DAG for covariate selection in analyses on prenatal exposure to endocrine conditions and ADHD risk [103–105].

## Data Availability

Data from the Norwegian Mother, Father and Child Cohort Study and the Medical Birth Registry of Norway used in this study are managed by the national health register holders in Norway (Norwegian Institute of Public Health) and can be made available to researchers, provided approval from the Regional Committees for Medical and Health Research Ethics (REC), compliance with the EU General Data Protection Regulation (GDPR) and approval from the data owners. The consent given by the participants does not open for storage of data on an individual level in repositories or journals. Researchers who want access to data sets for replication should apply through helsedata.no. Access to data sets requires approval from The Regional Committee for Medical and Health Research Ethics in Norway and an agreement with MoBa.

## Abbreviations

ADHD: Attention-deficit/hyperactivity disorder
MoBa: The Norwegian Mother, Father, and Child Cohort Study
MBRN: The Medical Birth Registry of Norway
NPR: The Norwegian Patient Registry
IgE: Immunoglobulin E
IgG: Immunoglobulin G
CD: Crohn’s disease
UC: Ulcerative colitis
RA: Rheumatoid arthritis
AS: Ankylosing spondylitis
SLE: Systemic lupus erythematosus
FMS: Fibromyalgia syndrome
T1D: Type 1 diabetes
T2D: Type 2 diabetes
GDM: Gestational diabetes mellitus
