# Supplementary material for: Maternal immune-mediated conditions and ADHD risk in offspring
Source: BMC Med. 2025 Jul 1;23:348. doi: 10.1186/s12916-025-04227-3 (PMC12210767; doi:10.1186/s12916-025-04227-3)
Supplement: Supplementary file 1 — Additional file 1: This file includes details on exposure data (Table S1), covariate selection and associations between covariates and between covariates and exposure variables (Tables S2 and S3), covariates included in different Dagitty models (DAGs) (Table S4), DAGs that forms the basis of the minimal sufficient adjustment sets of covariates for each of the exposure categories (Figs. S1–S10), and information regarding the handling of missing data. In addition, this contains descriptive information on exposed and unexposed cases as well as missing data for the main analyses (Tables S5 and S6). Additional analyses conducted on unimputed data are also included in the file (Table S7), as well as more detailed statistics from the negative control analyses (Table S8) and results from the sensitivity analyses (Tables S9 and S10). Table S1 Health conditions reported in questionnaires at specific times during pregnancy. Table S2 Associations between considered covariates and exposure variables. Table S3 Associations between covariates. Table S4 Covariates included in Dagitty models and minimum sufficient adjustment sets for each specific analysis. Table S5 Descriptive statistics for dataset analyzing maternal immune-mediated conditions in pregnancy and ADHD in offspring with Cox proportional hazards. Table S6 Descriptive statistics for dataset analyzing maternal diabetes in pregnancy and ADHD in offspring with Cox proportional hazards. Table S7 Associations between maternal immune-mediated conditions during pregnancy and ADHD in offspring examined with Cox proportional hazard analyses. Table S8 Associations between maternal gestational immune-mediated conditions and offspring ADHD diagnoses compared with the associations with paternal immune-mediated conditions, mutually adjusted for each other. Table S9 Sensitivity analyses for use of folic acid between 4 weeks before pregnancy and 8 weeks of gestation. Table S10 Analyses stratified by the specific immune-mediated conditi [file 12916_2025_4227_MOESM1_ESM.docx]

# Additional file 1

# Methods

## Maternal and Paternal Immune-Mediated Conditions

Questions on immune-mediated conditions differed slightly between maternal and paternal questionnaires. Mothers reported a few more types of immune-mediated conditions that fathers did (see Table S1 below), and the maternal exposure groups included in negative control designs were therefore adapted accordingly, so that included exposure variables were comparable between mothers and fathers. Paternal reports of diabetes were also not as specific as maternal reports, so in the negative control analysis all types of diabetes (potentially including type 2 diabetes as well as type 1) were included in both maternal and paternal exposure groups to achieve comparable conditions.

As some data (asthma and allergies) were collected at multiple time points during pregnancy, each instance increased the likelihood of detecting a condition. Hence, the reliability of this information may be higher than the information regarding conditions reported only once.

## Covariates

To ensure that covariate selection was grounded in existing knowledge of relevant causal pathways we started by selecting potential covariates based on previous studies. The covariates considered for inclusion were; mother's age, parity, and BMI, child's birth year, season of birth, area of residence, parental educational attainment and relationship status, mother's smoking and alcohol use before pregnancy, and mother's previous mental disorders and self‐reported ADHD symptoms (measured by the 6‐item World Health Organization adult ADHD self‐report scale screener (103)). These were all tested for associations with each of the exposure variables as well as the outcome variable to recognize potential confounding factors. All variables except maternal BMI were associated with the outcome ADHD variable. Table S2 below displays associations between the remaining covariates and exposure variables.

Associations between covariates are displayed in Table S3 below.

For each analysis, all covariates that were significantly associated with both the exposure and outcome were included in a Dagitty model to define the minimal sufficient adjustment sets of covariates for the specific analysis (104). Tables S2-3 (above) and figures S1-10 (below) provides more details about considered and included covariates and their associations.

Child's birth year, parental educational attainment and relationship status, mother's age, parity, smoking and alcohol use before pregnancy, and previous mental disorders and self‐reported ADHD symptoms were variables included in Dagitty models. Table S4 shows an overview of which variables were included in Dagitty models, and which of these were included in the minimum sufficient adjustment sets calculated by the Dagitty program.

Maternal hypertensive diseases during pregnancy, the child’s Apgar score, size for gestational age, and gestational age at birth, were thought to be on the causal path for the association between exposure and outcome and were therefore not included as potential confounders in the analyses.

Figures S1-S10 shows the Directed Acyclic Graphs (DAGs) that forms the basis of the minimal sufficient adjustment sets of covariates for each of the exposure categories.

**Figure S1 Directed Acyclic Graph (DAG) for Covariate Selection in Analyses on Prenatal Exposure to Asthma, Allergy, and Atopic Conditions and ADHD Risk**


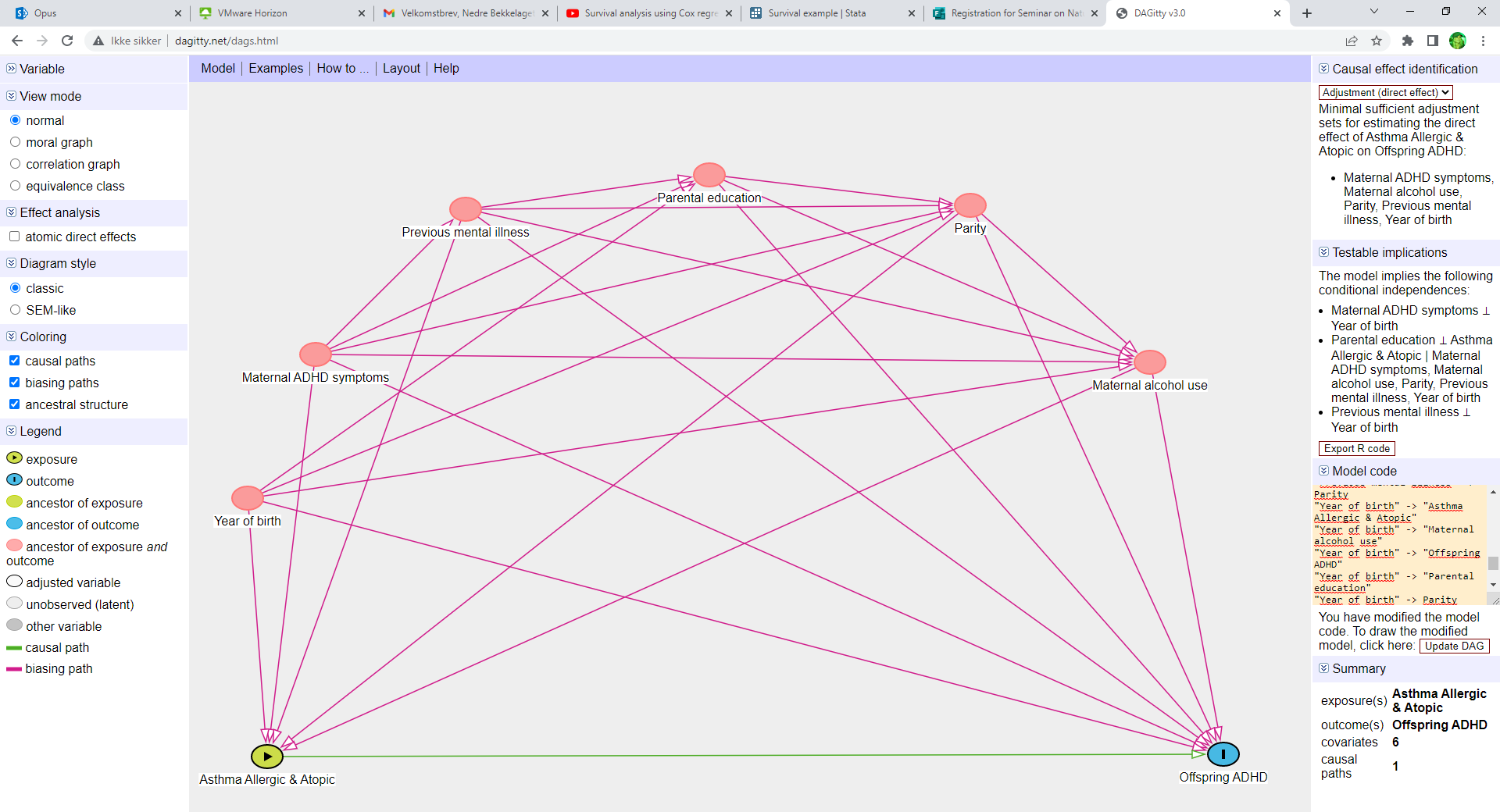


Minimal sufficient adjustment sets for estimating the direct effect of maternal asthma allergic atopic disorders on offspring ADHD: maternal ADHD symptoms, maternal alcohol use, parity, previous mental illness, year of birth.

**Figure S2 Directed Acyclic Graph (DAG) for Covariate Selection in Analyses on Prenatal Exposure to Autoimmune and inflammatory conditions and ADHD Risk**


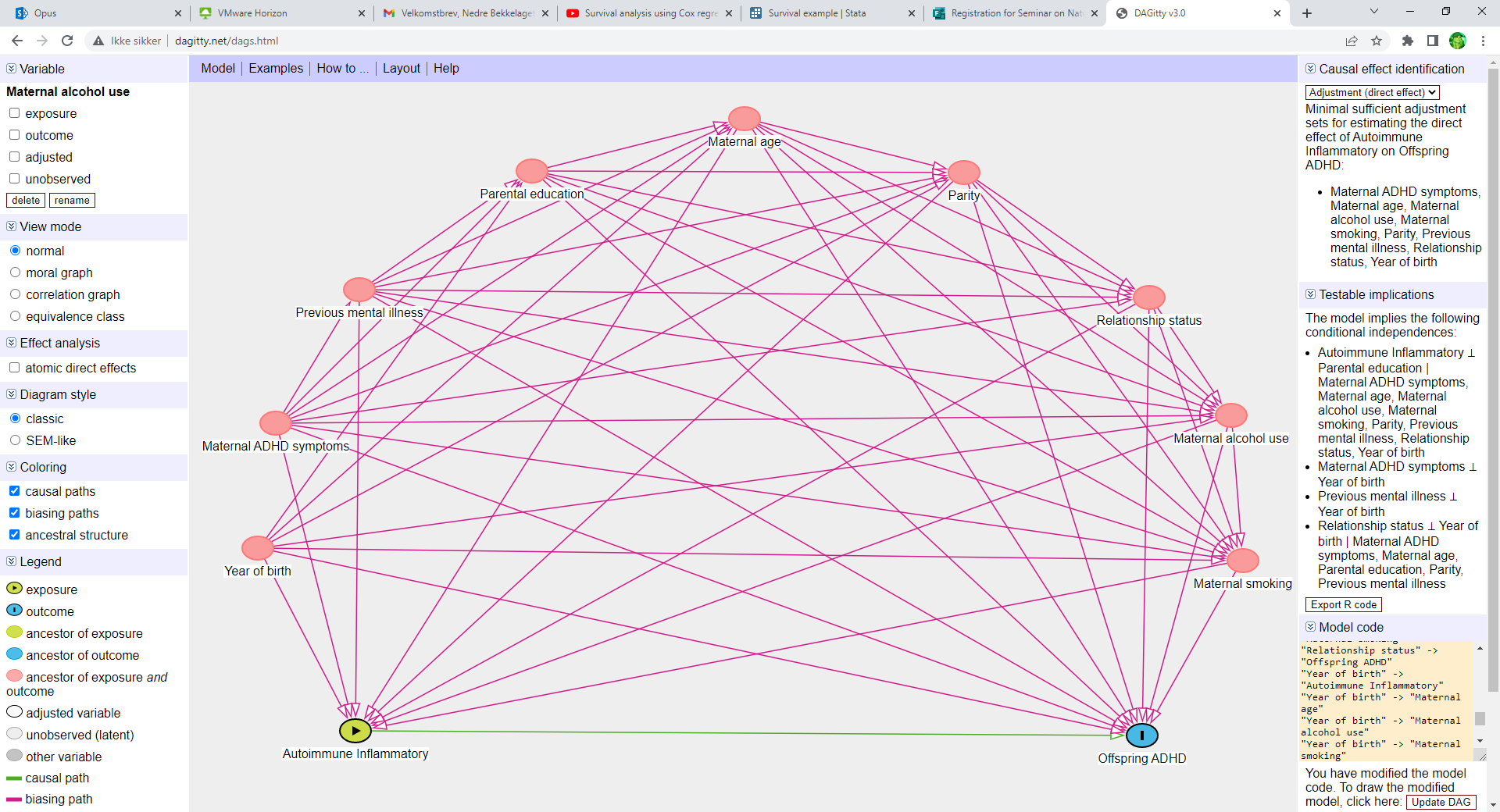


Minimal sufficient adjustment sets for estimating the direct effect of maternal autoimmune/inflammatory disorders on offspring ADHD: maternal ADHD symptoms, maternal age, maternal alcohol use, maternal smoking, parity, previous mental illness, relationship status, year of birth.

**Figure S3 Directed Acyclic Graph (DAG) for Covariate Selection in Analyses on Prenatal Exposure to Asthma and ADHD Risk**


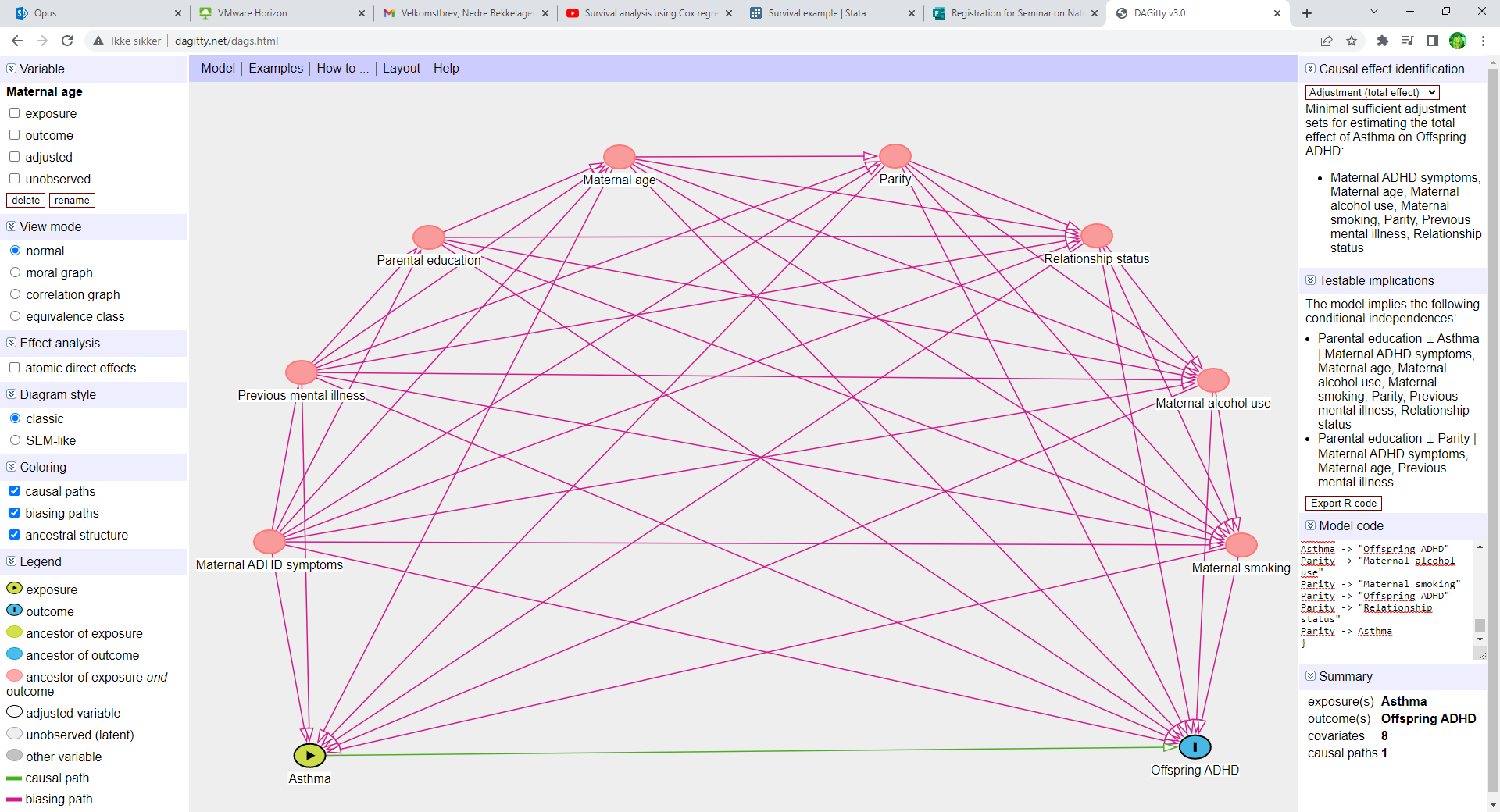


Minimal sufficient adjustment sets for estimating the direct effect of maternal asthma on offspring ADHD: maternal ADHD symptoms, maternal age, maternal alcohol use, maternal smoking, parity, previous mental illness, relationship status.

**Figure S4 Directed Acyclic Graph (DAG) for Covariate Selection in Analyses on Prenatal Exposure to Allergies and ADHD Risk**


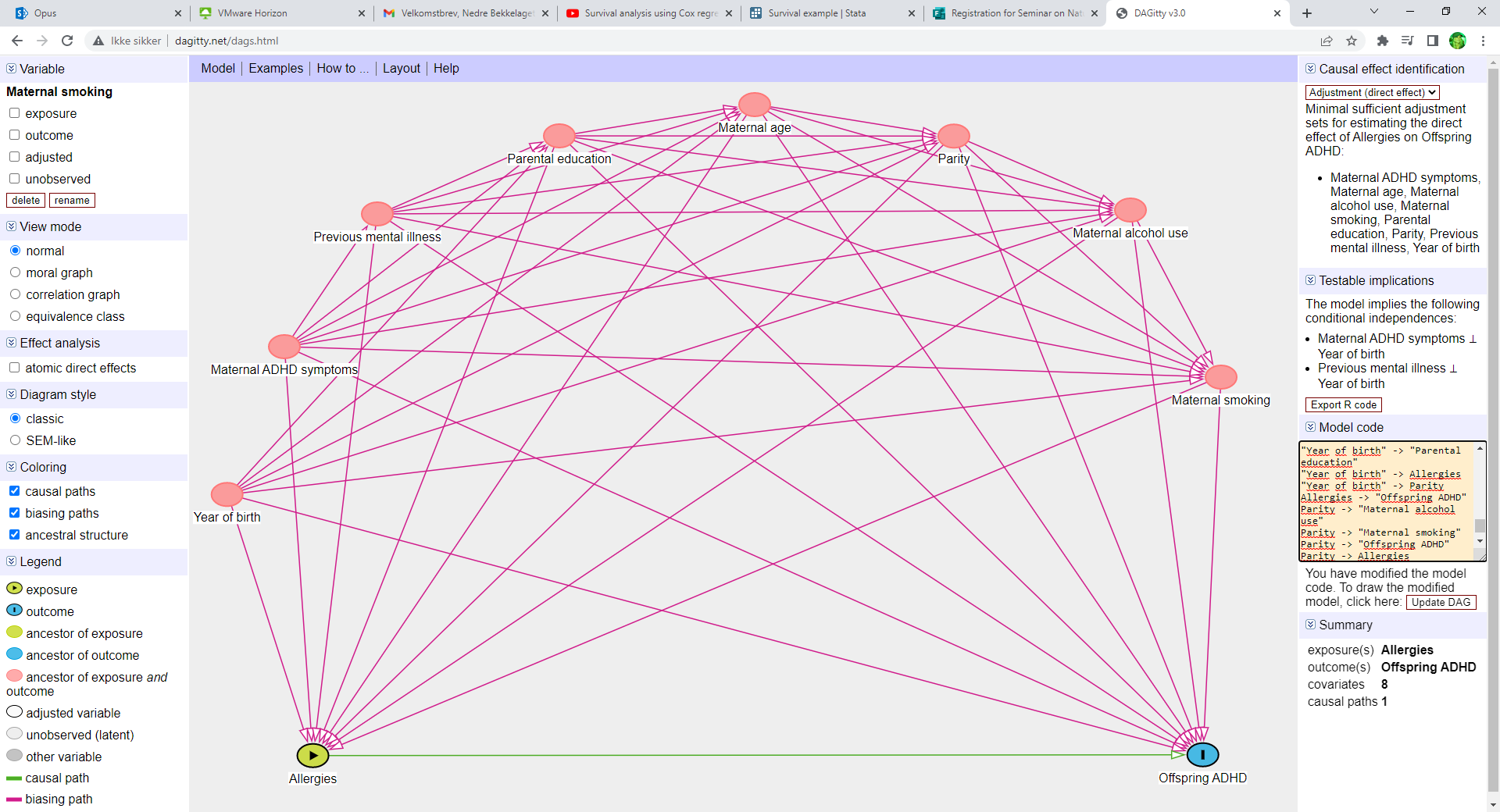


Minimal sufficient adjustment sets for estimating the direct effect of maternal allergies on offspring ADHD: maternal ADHD symptoms, maternal age, maternal alcohol use, maternal smoking, parental education, parity, previous mental illness, year of birth.

**Figure S5 Directed Acyclic Graph (DAG) for Covariate Selection in Analyses on Prenatal Exposure to Eczema and ADHD Risk**


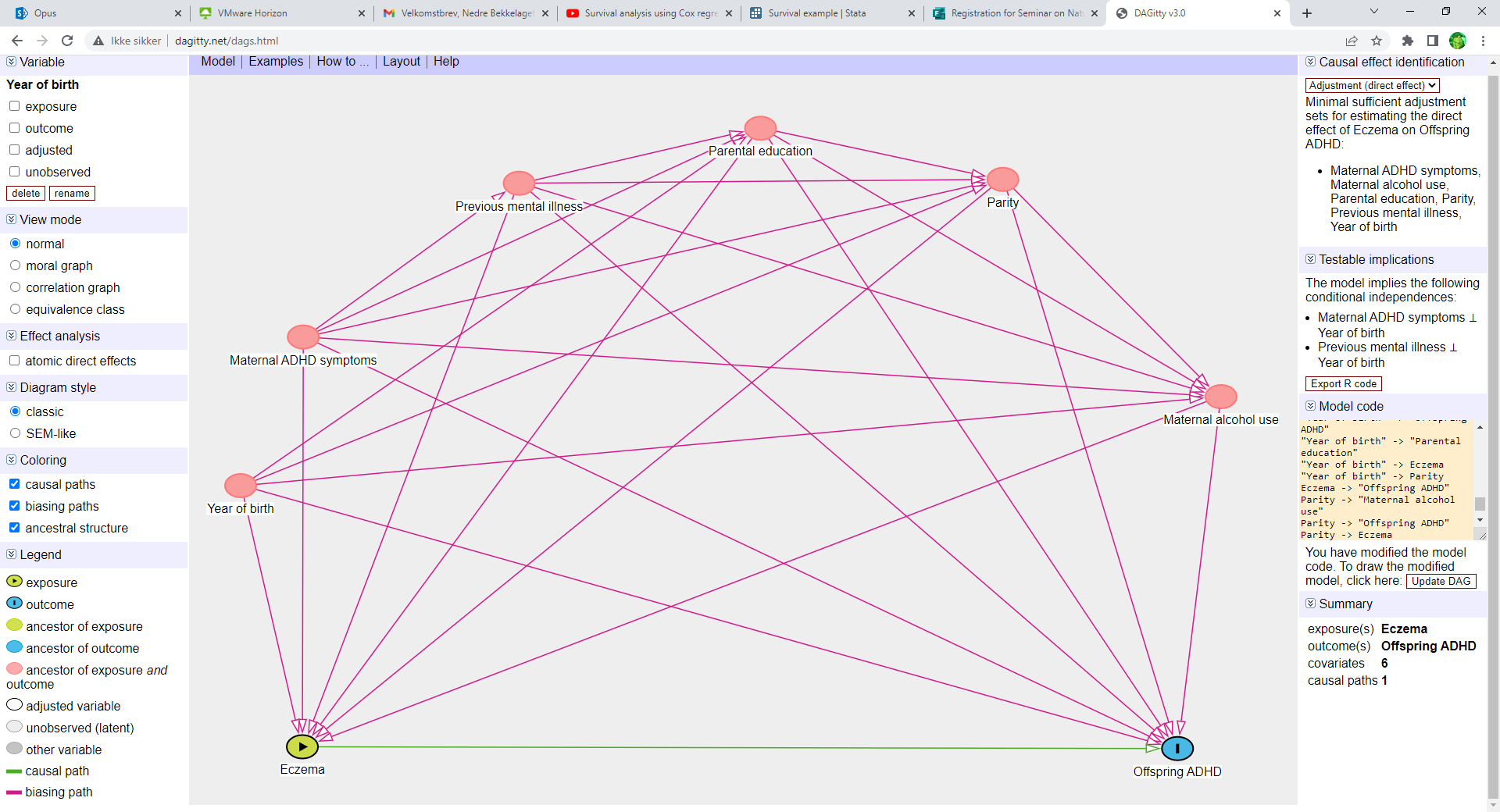


Minimal sufficient adjustment sets for estimating the direct effect of maternal eczema on offspring ADHD: maternal ADHD symptoms, maternal alcohol use, parental education, parity, previous mental illness, year of birth.

**Figure S6 Directed Acyclic Graph (DAG) for Covariate Selection in Analyses on Prenatal Exposure to Urticaria/Hives and ADHD Risk**


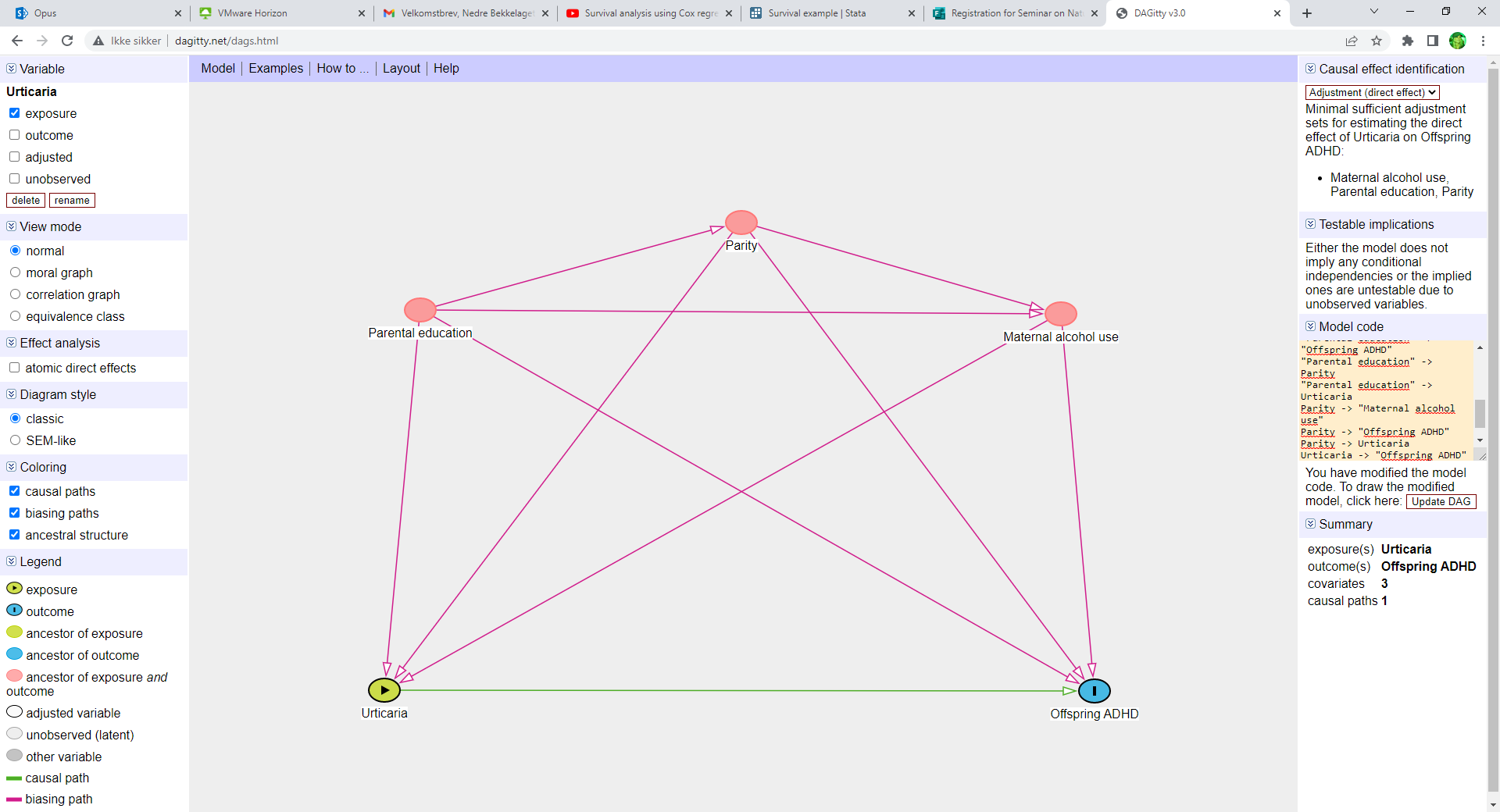


Minimal sufficient adjustment sets for estimating the direct effect of maternal urticaria on offspring ADHD: maternal alcohol use, parental education, parity.

**Figure S7 Directed Acyclic Graph (DAG) for Covariate Selection in Analyses on Prenatal Exposure to Psoriasis and ADHD Risk**


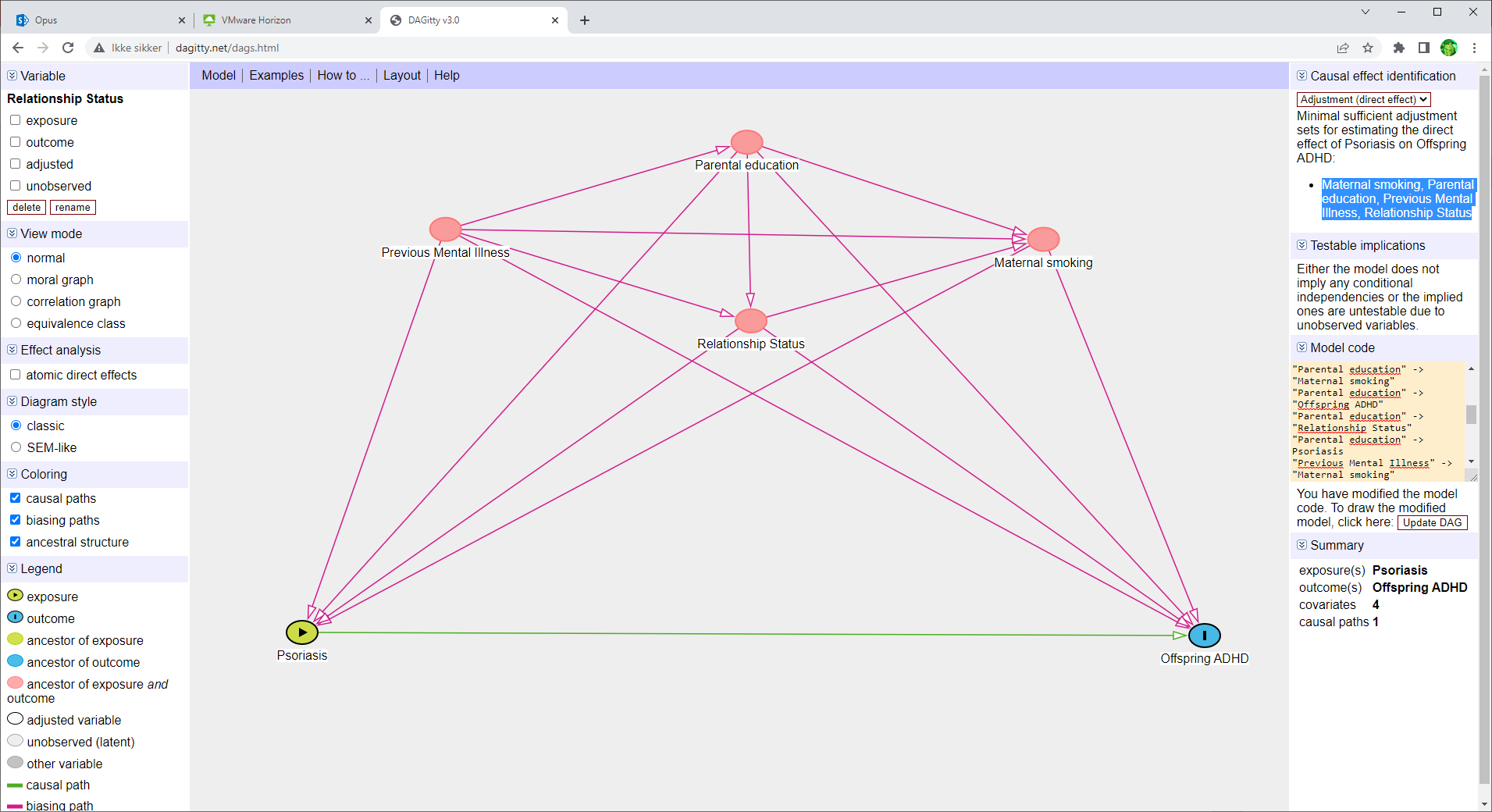


Minimal sufficient adjustment sets for estimating the direct effect of maternal urticaria on offspring ADHD: maternal smoking, parental education, previous mental illness, relationship status.

**Figure S8 Directed Acyclic Graph (DAG) for Covariate Selection in Analyses on Prenatal Exposure to Gastrointestinal Conditions and ADHD Risk**


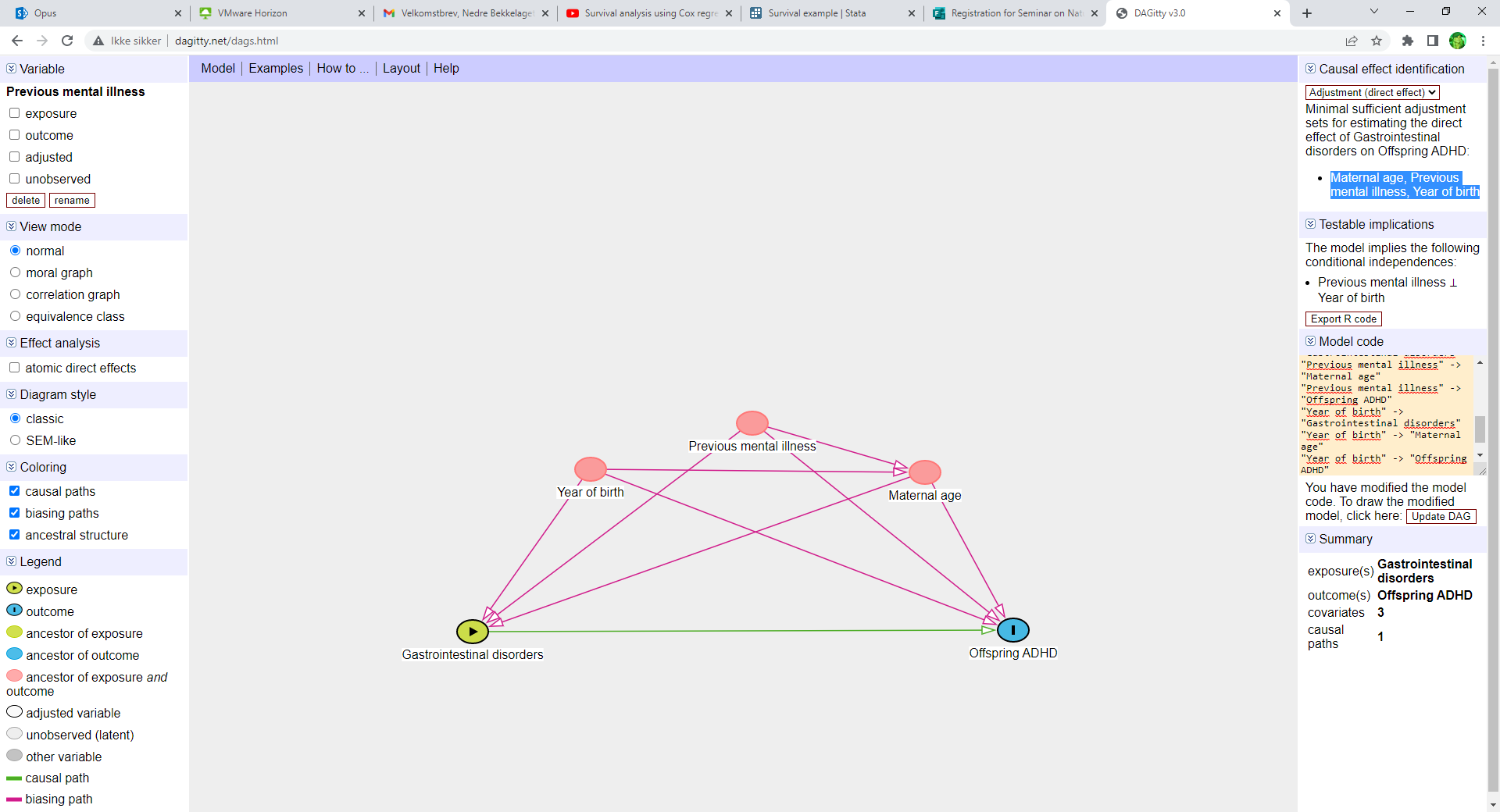


Minimal sufficient adjustment sets for estimating the direct effect of maternal UC/CD on offspring ADHD: maternal age, previous mental illness, year of birth.

**Figure S9 Directed Acyclic Graph (DAG) for Covariate Selection in Analyses on Prenatal Exposure to Rheumatologic Musculoskeletal Conditions and ADHD Risk**


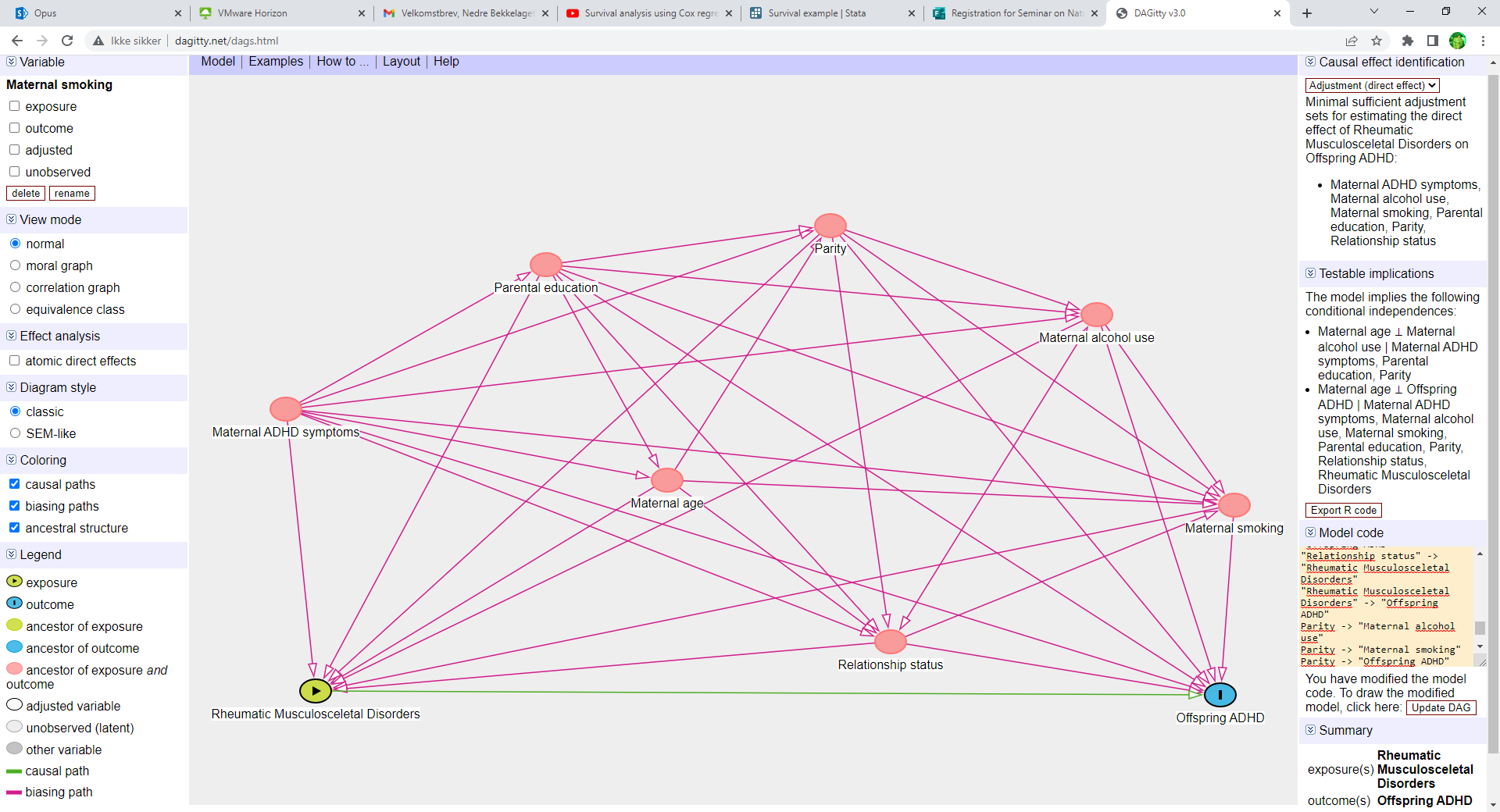


Minimal sufficient adjustment sets for estimating the direct effect of maternal rheumatologic musculoskeletal disorders on offspring ADHD: maternal ADHD symptoms, maternal alcohol use, maternal smoking, parental education, parity, relationship status.

**Figure S10 Directed Acyclic Graph (DAG) for Covariate Selection in Analyses on Prenatal Exposure to Endocrine Conditions and ADHD Risk**


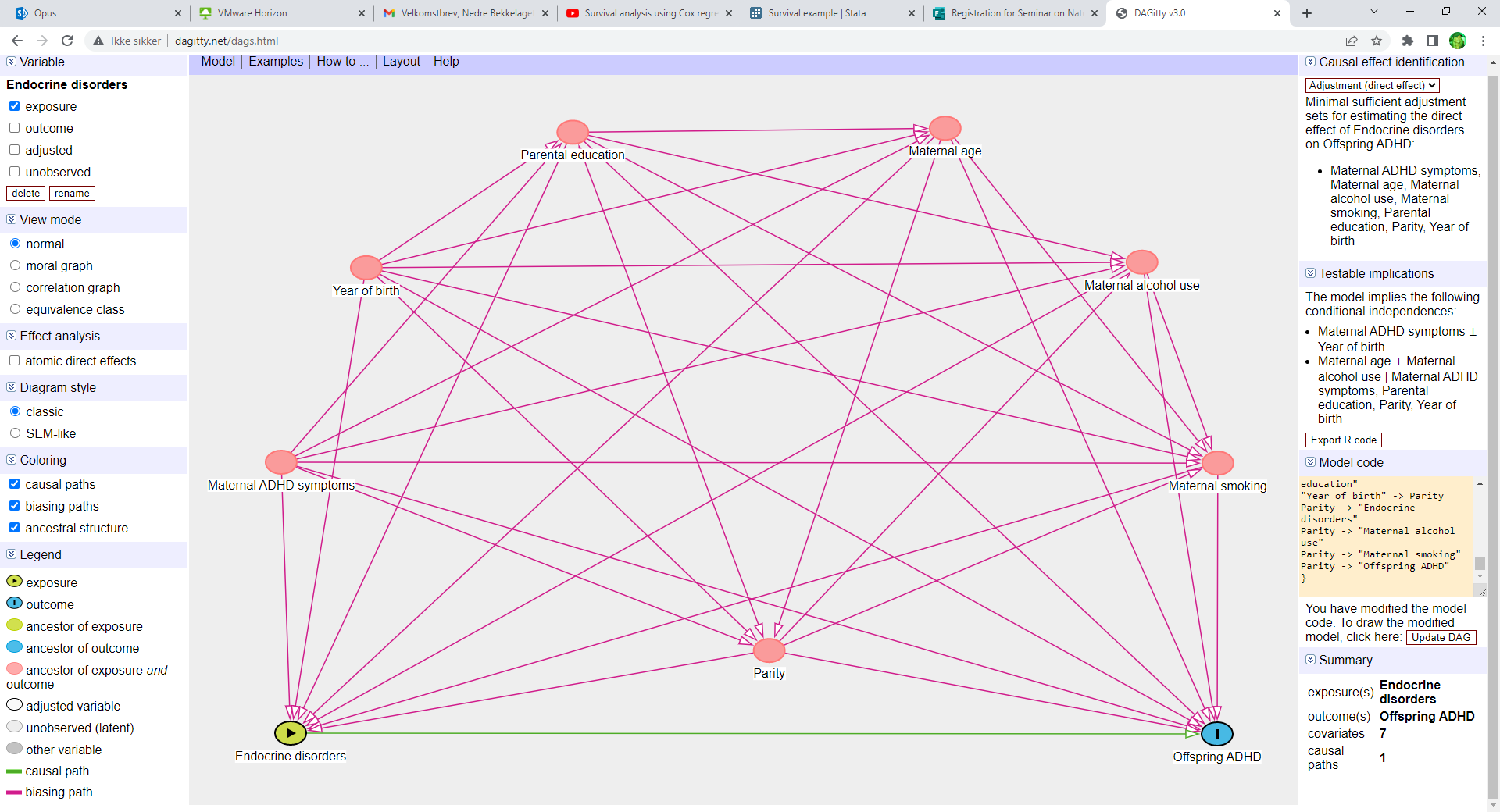


Minimal sufficient adjustment sets for estimating the direct effect of maternal diabetes on offspring ADHD: maternal ADHD symptoms, maternal age, maternal alcohol use, maternal smoking, parental education, parity, year of birth.

## Missing data

In 49.22% of pregnancies, information on maternal ADHD symptoms was missing. Additionally, parental educational attainment had 14.11% missing data, parental relationship status 9.91%, smoking habits 11.20%, and alcohol use before pregnancy 15.98%. Assuming these values were missing at random, we applied multiple imputation using the Markov chain Monte Carlo (MCMC) method, creating five datasets. The imputation model included all covariates alongside the exposure and outcome variables. Results from analyses on these data sets are presented in the main document.

Furthermore, analyses on available data without imputation were conducted to verify findings consistency. These results are presented below in table S7.

# Results

## Descriptives

Table S5 and S6 show some details on exposed and unexposed cases as well as missing data for analyses of offspring ADHD risk after prenatal exposures to different immune-mediated conditions in mothers (results are presented in the Main file: Table 2 and Table 3). Displayed are the number of exposed and unexposed cases for each type or category of exposure, total number of mother-child pairs included in analysis, the number of cases excluded from analysis due to missing data on maternal immune-mediated conditions.

| **TABLE S5 Descriptive Statistics for Dataset Analyzing Maternal Immune-Mediated Conditions in Pregnancy and ADHD in Offspring with Cox Proportional Hazards** | | | | | | |
| --- | --- | --- | --- | --- | --- | --- |
|  |  | No, of cases^a^ | Cases  excluded^b^ | Cases missing^c^ | *n*^d^ | Total *n*^e^ |
| Asthma/Allergic/Atopic Conditions | Unexposed | 68024 | 14623 | 283 | 89364 | 104270 |
|  | Exposed | 21340 |  |  |  |  |
| Autoimmune/Inflammatory Conditions | Unexposed | 94993 | 1516 | 283 | 102471 | 104270 |
|  | Exposed | 7478 |  |  |  |  |
|  |  |  |  |  |  |  |
| Asthma | Unexposed | 94991 | 3604 | 584 | 100082 | 104270 |
|  | Exposed | 5091 |  |  |  |  |
| Any Allergy (pollen, animal, other) | Unexposed | 75965 | 11329 | 558 | 92383 | 104270 |
|  | Exposed | 16418 |  |  |  |  |
| Atopic Eczema | Unexposed | 97079 | 2825 | 581 | 100864 | 104270 |
|  | Exposed | 3785 |  |  |  |  |
| Urticaria/Hives | Unexposed | 99735 | 3210 | 577 | 100483 | 104270 |
|  | Exposed | 748 |  |  |  |  |
| Psoriasis | Unexposed | 101031 | 1018 | 588 | 102664 | 104270 |
|  | Exposed | 1633 |  |  |  |  |
| Gastrointestinal Conditions | Unexposed | 102614 | 292 | 591 | 103387 | 104270 |
|  | Exposed | 773 |  |  |  |  |
| Rheumatologic/Musculoskeletal Conditions | Unexposed | 102064 | 558 | 591 | 103121 | 104270 |
|  | Exposed | 1057 |  |  |  |  |
| Endocrine Conditions | Unexposed | 99536 | 2049 | 589 | 101632 | 104270 |
|  | Exposed | 2096 |  |  |  |  |
| ^a^ No of exposed and unexposed cases | | | | | | |
| ^b^ Cases excluded due to condition being present before but not during pregnancy | | | | | | |
| ^c^ Cases excluded from analysis due to missing data | | | | | | |
| ^d^ No of pairs of mothers and children eligible for analysis  ^e^ No of pairs of mothers and children in total sample | | | | | | |

| **Table S6 Descriptive Statistics for Dataset Analyzing Maternal Diabetes in Pregnancy and ADHD in Offspring with Cox Proportional Hazards** | | | | |
| --- | --- | --- | --- | --- |
|  |  | No. of cases^a^ | Cases of missing data | *n*^b^ |
| Diabetes 1 | Unexposed | 102084 | 1885 | 102385 |
|  | Exposed | 301 |  |  |
| Diabetes 2 | Unexposed | 102084 | 2037 | 102233 |
|  | Exposed | 149 |  |  |
| Gestational diabetes | Unexposed | 102084 | 1211 | 103059 |
|  | Exposed | 975 |  |  |
| ^a^ No of exposed and unexposed cases | | | |  |
| ^b^ No of pairs of mothers and children | | | |  |

## Main analyses without imputations

Due to significant missing data on maternal ADHD symptoms in 49.22% of pregnancies, we conducted multiple imputations to retain statistical power. However, with such a high proportion of missing values, imputations may become biased and less reliable. To assess the robustness of our initial findings, we conducted additional analyses both including and excluding the influence of this covariate. Table S7 presents results from analyses on the original available sample, without imputations for missing data. The first set of columns show results from unadjusted main analyses; subsequent columns present analyses adjusted for covariate sets, one excluding and one including maternal ADHD symptoms as a covariate. Although samples were considerably reduced due to high missing rates for certain covariates, specifically maternal ADHD symptoms, the results largely remained consistent, though with some effect sizes showing some increase or decrease.

| **TABLE S7 Associations Between Maternal Immune-Mediated Conditions During Pregnancy and ADHD in Offspring Examined with Cox Proportional Hazard Analyses** | | | | | | | | | | | | | | | |
| --- | --- | --- | --- | --- | --- | --- | --- | --- | --- | --- | --- | --- | --- | --- | --- |
|  | Crude | | | | | Adjusted^a^ | | | | | Adjusted^b^ | | | | |
|  | Hazard Ratio | SE | 95% CI | *P* | n^c^ | Hazard Ratio | SE | 95% CI | *P* | n^c^ | Hazard Ratio | SE | 95% CI | *P* | n^c^ |
| Asthma/Allergic/Atopic Conditions | 1,20 | 0,05 | 1,11-1,30 | <,001 | 89364 | 1,17 | 0,05 | 1,07-1,27 | <,001 | 73500 | 1,22 | 0,07 | 1,09-1,37 | 0,001 | 42118 |
| Autoimmune/Inflammatory Conditions | 1,31 | 0,08 | 1,17-1,47 | <,001 | 102471 | 1,33 | 0,08 | 1,17-1,50 | <,001 | 84893 | 1,41 | 0,12 | 1,20-1,66 | <,001 | 48829 |
|  |  |  |  |  |  |  |  |  |  |  |  |  |  |  |  |
| Asthma | 1,61 | 0,10 | 1,42-1,83 | <,001 | 100383 | 1,38 | 0,10 | 1,20-1,59 | <,001 | 82971 | 1,57 | 0,15 | 1,30-1,89 | <,001 | 47780 |
| Any Allergy (pollen, animal, other) | 1,14 | 0,05 | 1,04-1,24 | 0,004 | 92658 | 1,17 | 0,06 | 1,06-1,28 | 0,001 | 72352 | 1,20 | 0,08 | 1,05-1,36 | 0,006 | 41684 |
| Atopic Eczema | 1,06 | 0,09 | 0,89-1,26 | 0,525 | 101162 | 1,13 | 0,10 | 0,95-1,36 | 0,176 | 80245 | 1,13 | 0,14 | 0,89-1,45 | 0,311 | 46232 |
| Urticaria/Hives | 1,11 | 0,21 | 0,77-1,60 | 0,577 | 100777 | 1,17 | 0,23 | 0,80-1,71 | 0,422 | 79873 | 0,97 | 0,28 | 0,55-1,72 | 0,921 | 46043 |
| Psoriasis | 1,19 | 0,15 | 0,93-1,51 | 0,163 | 102969 | 1,09 | 0,14 | 0,85-1,41 | 0,482 | 86378 | 1,25 | 0,21 | 0,90-1,74 | 0,185 | 49041 |
| Gastrointestinal Conditions | 1,20 | 0,23 | 0,83-1,73 | 0,340 | 103695 | 1,28 | 0,24 | 0,89-1,85 | 0,186 | 103695 | 1,43 | 0,33 | 0,91-2,24 | 0,123 | 52748 |
| Rheumatologic/Musculoskeletal Conditions | 1,80 | 0,23 | 1,41-2,31 | <,001 | 103121 | 1,84 | 0,24 | 1,43-2,37 | <,001 | 81604 | 1,77 | 0,32 | 1,24-2,53 | 0,002 | 47155 |
| Endocrine Conditions | 1,32 | 0,14 | 1,07-1,63 | 0,010 | 101938 | 1,31 | 0,16 | 1,04-1,65 | 0,023 | 80683 | 1,24 | 0,21 | 0,90-1,72 | 0,177 | 46591 |
| Note: Separate analyses were performed for each of the exposure variables. CI, Confidence interval. The α level was set to .01 to indicate significant associations. | | | | | | | | | | | | | | | |
| ^a^ Each analyses had specific adjustment sets of covariates. In these analyses maternal ADHD symptoms were not included as covariates. Adjustment sets were the following: Asthma/Allergic/Atopic Conditions: child's birth year, mother's parity, alcohol use before pregnancy, and previous mental disorders; Autoimmune/Inflammatory Conditions: child's birth year, parental relationship status, mother's age, parity, smoking and alcohol use before pregnancy, and previous mental disorders; Asthma: parental relationship status, mother's age, parity, smoking and alcohol use before pregnancy, and previous mental disorders; Any Allergy: child's birth year, parental educational attainment, mother's age, parity, smoking and alcohol use before pregnancy, and previous mental disorders; Atopic Eczema: child's birth year, parental educational attainment, mother's parity, alcohol use before pregnancy, and previous mental disorders; Urticaria/Hives: parental educational attainment, mother's parity and alcohol use before pregnancy; Psoriasis: parental educational attainment and relationship status, mother's smoking and previous mental disorders; Gastrointestinal Conditions: child's birth year, mother's age and previous mental disorders; Rheumatologic/Musculoskeletal Conditions: parental educational attainment and relationship status, mother's parity, and smoking and alcohol use before pregnancy; Endocrine Conditions (T1D and hyper/hypothyroidism): child's birth year, parental educational attainment, mother's age, parity, and smoking and alcohol use before pregnancy. | | | | | | | | | | | | | | | |
| ^b^ These analyses adjusted for self-reported maternal ADHD symptoms in addition to the adjustment sets of covariates created for the specific exposures. | | | | | | | | | | | | | | | |
| ^c^ No of pairs of mothers and children. | | | | | | | | | | | | | | | |

## Negative control analyses

The results of the negative control analyses are presented in a forest plot in the Main file: Figure 2. Table S8 presents additional details about the statistics of these analyses.

| **TABLE S8 Associations Between Maternal Gestational Immune-Mediated Conditions and Offspring ADHD Diagnoses Compared with the Associations with Paternal Immune-Mediated Conditions, Mutually Adjusted For Each Other** | | | | | | | | | | | |
| --- | --- | --- | --- | --- | --- | --- | --- | --- | --- | --- | --- |
|  | Crude^a^ | | | | | Adjusted^b^ | | | | |  |
|  | Hazard Ratio | *P* | 95% CI | X2 Value | *P* Value X2 Test | Hazard Ratio | *P* | 95% CI | X2 Value | *P* Value X2 Test | *n* |
| Asthma mother | 1,45 | <,001 | 1,23-1,71 | 0,69 | 0,406 | 1,33 | 0,001 | 1,13-1,58 | 0,26 | 0,607 | 68248 |
| Asthma father | 1,32 | <,001 | 1,15-1,52 |  |  | 1,26 | 0,001 | 1,10-1,45 |  |  | 68248 |
| Pollen allergy mother | 1,22 | 0,001 | 1,09-1,37 | 30,98 | <,001 | 1,26 | <,001 | 1,12-1,41 | 26,49 | <,001 | 64167 |
| Pollen allergy father | 0,76 | <,001 | 0,67-0,86 |  |  | 0,81 | 0,001 | 0,72-0,92 |  |  | 64167 |
| Atopic Eczema mother | 1,16 | 0,147 | 0,95-1,42 | 1,51 | 0,220 | 1,20 | 0,078 | 0,98-1,46 | 1,06 | 0,303 | 68814 |
| Atopic Eczema father | 0,98 | 0,801 | 0,81-1,17 |  |  | 1,04 | 0,704 | 0,86-1,24 |  |  | 68814 |
| Urticaria/hives mother | 1,05 | 0,843 | 0,67-1,65 | 0,07 | 0,797 | 1,05 | 0,837 | 0,67-1,65 | 0,02 | 0,879 | 68512 |
| Urticaria/hives father | 0,98 | 0,840 | 0,83-1,17 |  |  | 1,01 | 0,913 | 0,85-1,20 |  |  | 68512 |
| Psoriasis mother | 1,34 | 0,036 | 1,02-1,76 | 0,96 | 0,326 | 1,24 | 0,114 | 0,95-1,63 | 0,40 | 0,526 | 70216 |
| Psoriasis father | 1,13 | 0,240 | 0,92-1,38 |  |  | 1,12 | 0,290 | 0,91-1,36 |  |  | 70216 |
| CD / UC mother | 1,89 | 0,006 | 1,20-2,98 | 3,09 | 0,079 | 1,95 | 0,004 | 1,23-3,09 | 3,75 | 0,053 | 70820 |
| CD / UC father | 1,20 | 0,124 | 0,95-1,50 |  |  | 1,18 | 0,160 | 0,94-1,48 |  |  | 70820 |
| Rheumatoid arthritis / Ankylosing spondylitis mother | 1,48 | 0,075 | 0,96-2,27 | 1,61 | 0,204 | 1,38 | 0,139 | 0,90-2,13 | 1,41 | 0,235 | 70745 |
| Rheumatoid arthritis / Ankylosing spondylitis father | 0,97 | 0,908 | 0,61-1,55 |  |  | 0,93 | 0,773 | 0,59-1,49 |  |  | 70745 |
| Any diabetes mother | 1,45 | 0,018 | 1,07-1,97 | 1,75 | 0,185 | 1,39 | 0,037 | 1,02-1,90 | 1,79 | 0,182 | 70870 |
| Any diabetes father | 1,01 | 0,953 | 0,65-1,57 |  |  | 0,97 | 0,884 | 0,62-1,50 |  |  | 70870 |
| CI, confidence interval.  ^a^ Maternal gestational immune-mediated conditions and paternal immune-mediated conditions mutually adjusted for each other, but not adjusted for covariates.  ^b^ Maternal smoking and the negative controls mutually adjusted for each other and for the following covariates: Asthma: parental relationship status, mother's age, parity, smoking and alcohol use before pregnancy, previous mental disorders and self‐reported ADHD symptoms; Pollen allergy: child's birth year, parental educational attainment, mother's age, parity, smoking and alcohol use before pregnancy, previous mental disorders and self‐reported ADHD symptoms; Atopic eczema: child's birth year, parental educational attainment, mother's parity, alcohol use before pregnancy, previous mental disorders and self‐reported ADHD symptoms; Urticaria/hives: parental educational attainment, mother's parity and alcohol use before pregnancy; Psoriasis: parental educational attainment and relationship status, mother's smoking and previous mental disorders; CD/UC: child's birth year, mother's age and previous mental disorders; Rheumatoid arthritis/Ankylosing spondylitis: parental educational attainment and relationship status, mother's parity, smoking and alcohol use before pregnancy, and self‐reported ADHD symptoms; Any diabetes: child's birth year, parental educational attainment, mother's age, parity, smoking and alcohol use before pregnancy, and self‐reported ADHD symptoms. χ2 values were obtained from testing 3 null hypotheses that the association between maternal smoking and offspring ADHD diagnoses was equal to each of the 3 other associations (Wald tests).  Degrees of freedom = 1 in all tests | | | | | | | | | | | |

## Sensitivity analyses

Sensitivity analyses assessed the impact of folate use during pregnancy (from 4 weeks before pregnancy until 8 weeks of gestation), given its role in immune system balance (105). Cox regressions were performed for each immune-mediated condition, assessing main effects of the specific condition and of folate use, as well as the potential interaction effect between the two. In addition, Cox regressions were run within groups exposed to the immune-mediated condition, assessing the impact of folate use during pregnancy on the ADHD risk in this group. These results are presented in Table S9 below, with no interactions being significant.

| **Table S9 Sensitivity Analyses for Use of Folic Acid between 4 Weeks Before Pregnancy to 8 Weeks of Gestation.** | | | | | |
| --- | --- | --- | --- | --- | --- |
|  | Adjusted Hazard Ratio | SE | 95% CI | *P* | *n* |
| Asthma | 1,45 | 0,14 | 1,20-1,75 | <,001 | 100383 |
| Folic Acid | 1,04 | 0,13 | 0,82-1,33 | 0,737 |  |
| Asthma x Folic Acid | 0,97 | 0,12 | 0,76-1,25 | 0,831 |  |
| Asthma and No Folic Acid | 1,46 | 0,14 | 1.21-1,76 | <,001 | 37967 |
| Asthma and Folic Acid | 1,47 | 0,13 | 1,24-1,74 | <,001 | 62416 |
|  |  |  |  |  |  |
| Pollen Allergy | 1,17 | 0,10 | 0,99-1,39 | 0,067 | 95104 |
| Folic Acid | 1,1 | 0,11 | 0,91-1,33 | 0,336 |  |
| Pollen Allergy x Folic Acid | 0,91 | 0,10 | 0,74-1,12 | 0,363 |  |
| Pollen Allergy and No Folic Acid | 1,14 | 0,10 | 0.96-1,35 | 0,129 | 36543 |
| Pollen Allergy and Folic Acid | 1,28 | 0,08 | 1,14-1,44 | <,001 | 58561 |
|  |  |  |  |  |  |
| Atopic Eczema | 1,26 | 0,19 | 0,94-1,68 | 0,117 | 101162 |
| Folic Acid | 0,85 | 0,15 | 0,59-1,21 | 0,363 |  |
| Atopic Eczema x Folic Acid | 1,18 | 0,22 | 0,82-1,70 | 0,368 |  |
| Atopic Eczema and No Folic Acid | 1,23 | 0,18 | 0,92-1,64 | 0,169 | 38226 |
| Atopic Eczema and Folic Acid | 1,07 | 0,12 | 0,86-1,33 | 0,533 | 62936 |
|  |  |  |  |  |  |
| Urticaria | 1,2 | 0,38 | 0,64-2,22 | 0,569 | 100777 |
| Folic Acid | 0,86 | 0,34 | 0,40-1,85 | 0,697 |  |
| Urticaria x Folic Acid | 1,11 | 0,43 | 0,51-2,38 | 0,798 |  |
| Urticaria and No Folic Acid | 1,19 | 0,38 | 0,64-2,21 | 0,581 | 38175 |
| Urticaria and Folic Acid | 1,08 | 0,25 | 0,68-1,70 | 0,747 | 62602 |
|  |  |  |  |  |  |
| Psoriasis | 1,05 | 0,23 | 0,69-1,61 | 0,811 | 102969 |
| Folic Acid | 1,21 | 0,31 | 0,72-2,01 | 0,470 |  |
| Psoriasis x Folic Acid | 0,9 | 0,24 | 0,54-1,50 | 0,682 |  |
| Psoriasis and No Folic Acid | 1,03 | 0,22 | 0,67-1,57 | 0,890 | 38682 |
| Psoriasis and Folic Acid | 1,16 | 0,17 | 0,87-1,56 | 0,311 | 64287 |
|  |  |  |  |  |  |
| Crohn's Disease / Ulcerative Colitis (CD/UC) | 1,01 | 0,49 | 0,39-2,63 | 0,983 | 103783 |
| Folic Acid | 1,59 | 0,82 | 0,57-4,38 | 0,372 |  |
| CD/UC x Folic Acid | 0,59 | 0,31 | 0,21-1,63 | 0,309 |  |
| CD/UC and No Folic Acid | 0,96 | 0,47 | 0,37-2,50 | 0,941 | 38944 |
| CD/UC and Folic Acid | 1,72 | 0,42 | 1,07-2,78 | 0,026 | 64839 |
|  |  |  |  |  |  |
| Rheumatoid Arthritis / Ankylosing Spondylitis (RA/AS) | 1,45 | 0,41 | 0,83-2,51 | 0,188 | 103675 |
| Folic Acid | 0,95 | 0,34 | 0,47-1,92 | 0,876 |  |
| RA/AS x Folic Acid | 1,03 | 0,37 | 0,51-2,10 | 0,933 |  |
| RA/AS and No Folic Acid | 1,44 | 0,41 | 0,83-2,50 | 0,190 | 38897 |
| RA/AS and Folic Acid | 1,39 | 0,32 | 0,89-2,18 | 0,152 | 64778 |
|  |  |  |  |  |  |
| Diabetes 1 | 2,79 | 0,79 | 1,60-4,85 | <,001 | 102691 |
| Folic Acid | 0,75 | 0,29 | 0,36-1,59 | 0,456 |  |
| Diabetes 1 x Folic Acid | 1,25 | 0,48 | 0,59-2,63 | 0,564 |  |
| Diabetes 1 and No Folic Acid | 2,99 | 0,86 | 1,70-5,25 | <,001 | 38511 |
| Diabetes 1 and Folic Acid | 2,19 | 0,61 | 1,26-3,79 | 0,005 | 64180 |

Cox regressions were also performed for each immune-mediated condition, assessing potential effects of the medication used on offspring ADHD risk. Analyses were stratified by the presence or absence of the specific immune-mediated condition assessed, and since there were typically no medications used by unexposed participants, effects shown in Table S10 are effects of medication within the exposed group, with none of these effects being significant.

| **Table S10 Analyses Stratified by the Specific Immune-Mediated Condition Assessing whether Medications Alters the Risk of Developing ADHD among e.g. Asthmatics.** | | | | |
| --- | --- | --- | --- | --- |
|  | Adjusted | | | |
| Condition strata | Hazard Ratio | SE | 95% CI | *P* |
| Asthma | 1,16 | 0,14 | 0,91-1,47 | 0,224 |
| Allergy | 1,03 | 0,09 | 0,86-1,23 | 0,738 |
| Atopic eczema | 0,77 | 0,13 | 0,55-1,08 | 0,126 |
| Urticaria | 0,62 | 0,24 | 0,29-1,34 | 0,226 |
| Psoriasis | 1,38 | 0,34 | 0,85-2,24 | 0,195 |
| CD/UC | 0,62 | 0,28 | 0,26-1,49 | 0,282 |
| RA/AS | 1,26 | 0,50 | 0,58-2,74 | 0,554 |
| Diabetes Type 1 | 1,58 | 0,69 | 0,67-3,72 | 0,291 |
